# Context-dependent prediction of protein complexes by SiComPre

**DOI:** 10.1038/s41540-018-0073-0

**Published:** 2018-09-17

**Authors:** Simone Rizzetto, Petros Moyseos, Bianca Baldacci, Corrado Priami, Attila Csikász-Nagy

**Affiliations:** 1 The Microsoft Research—University of Trento Centre for Computational and Systems Biology (COSBI), Piazza Manifattura, 1, 38068 Rovereto, TN Italy; 2 School of Medical Sciences, UNSW Australia, Sydney, Australia; 3 Department of Computer Science, University of Pisa, Pisa, Italy; 4 Randall Centre for Cell and Molecular Biophysics, King’s College London, London, UK; 5 Faculty of Information Technology and Bionics, Pázmány Péter Catholic University, Budapest, Hungary

## Abstract

Most cellular processes are regulated by groups of proteins interacting together to form protein complexes. Protein compositions vary between different tissues or disease conditions enabling or preventing certain protein−protein interactions and resulting in variations in the complexome. Quantitative and qualitative characterization of context-specific protein complexes will help to better understand context-dependent variations in the physiological behavior of cells. Here, we present SiComPre 1.0, a computational tool that predicts context-specific protein complexes by integrating multi-omics sources. SiComPre outperforms other protein complex prediction tools in qualitative predictions and is unique in giving quantitative predictions on the complexome depending on the specific interactions and protein abundances defined by the user. We provide tutorials and examples on the complexome prediction of common model organisms, various human tissues and how the complexome is affected by drug treatment.

## Introduction

Most proteins are biologically active only when they serve as part of a protein complex and their identification is important to understand cellular functions. Protein complex assembly is limited by multiple constraints that need to be considered for protein complex predictions. A pair of interacting proteins has to be expressed in the same cell, within the same subcellular compartment at the same time. Moreover, high abundance of a protein can titrate away some or all of its binding partners, which will not be capable of interacting with other molecules. Binding sites are also a finite resource of a protein, limiting the number of possible simultaneous interactions. Even when multiple binding sites are available, it is not always possible for a protein to have many simultaneous interactions, since a bounded protein might make other binding sites inaccessible due to its size. Furthermore, cellular functions are dependent on the quantities of protein complexes,^1^ for instance, the number of ribosomes can affect cell growth rate.^2^ Unfortunately, we have very limited data on these. In order to study protein complexes, new experimental assays have been recently developed to reconstruct protein−protein interactions, protein complex composition, and stoichiometry coefficients within HeLa cell line.^3^ On the other hand, computational methods can now identify protein complexes from interaction networks.^4^ However, these methods cannot provide quantitative prediction of protein complexes yet. Solving this task for the whole proteome is complicated by the large scale of this system and requires high computational power. Computational approaches that aims to predict such information requires finding the right abstraction level to model protein complex formation. High resolution simulations can provide detailed information of a limited set of protein complexes,^5,6^ but cannot be applied to large-scale scenarios. To solve these issues, we previously designed SiComPre, a method to perform stochastic simulations of protein complexation and decomplexation reactions.^1^ Models were generated from a priori information: protein−protein interactions, domain−domain interactions, GO functional terms, and protein abundances. In a stochastic simulation (inspired by Gillespie MultiParticle algorithm^7^) we follow each individual molecule and track its binding and unbinding reactions as it diffuses through well-mixed subvolumes, where these reactions can occur. Domains were used as binding sites for a protein and in the case of a PPI the proteins that do not have any interacting domains, we added a pair of interacting fictitious domains only if the two proteins have a common “biological function” according to Gene Ontology (GO).^8^ Space is modeled as a two-dimensional grid where each lattice is a subvolume and proteins can diffuse across them at each timepoint according the Flick’s law. Between two timepoints an instance of Gillespie’s algorithm is executed for each subvolume which simulates complexation and decomplexation reactions between proteins present in the same subvolume. This approach allows us to simulate the competitive binding to a protein binding site and predict protein complexes specific for a condition corresponding to the protein abundance data used as input of the model. Finally, simulation results are postprocessed to obtain qualitative and quantitative prediction of protein complexes.

In the first version of the method, the simulation space was not corresponding to any biological meaning, each protein could have unlimited simultaneous binding partners and we could not provide a software tool for users without advanced computational skills. Here we present SiComPre 1.0, a software tool based on an updated version of our previously defined method. SiComPre 1.0 has an easy-to-use graphical user interface allowing the customization of all parameters and setting of the simulations. Furthermore, it provides a tool to compare complexomes simulated with various settings, enabling context-dependent prediction of protein complexes. Finally, we optimized the core simulation algorithm to increase computational performance on GPU.

## SiComPre 1.0

The goals of SiComPre 1.0 are to provide an accessible interface to all its features, provide the capability to compare results to investigate the effects of context-specific proteomics data on predictions and to improve the scores in predicting protein complexes.

### New processing pipeline

**Fig. 1:**
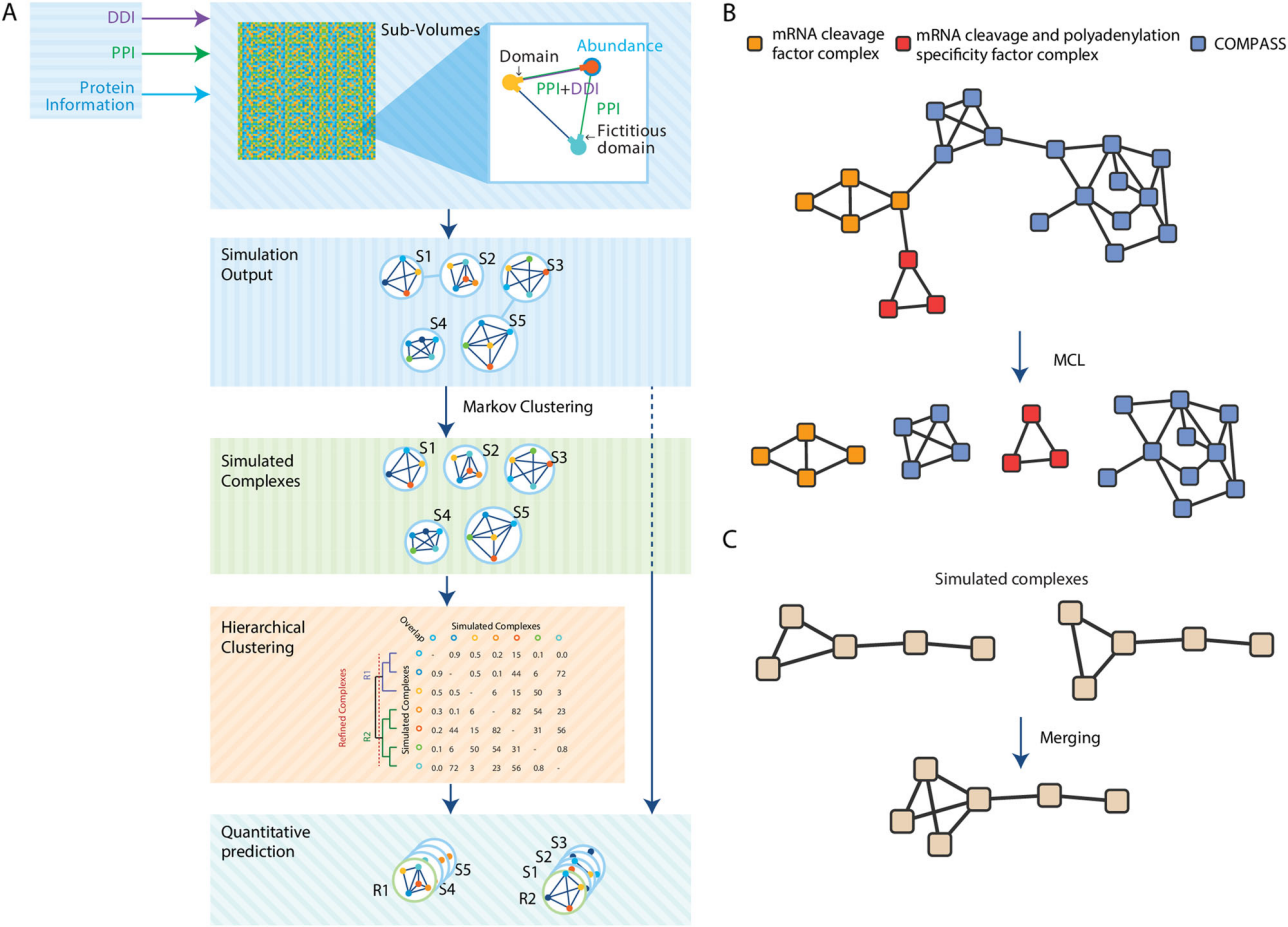
Overview of the pipeline. **a** The steps used by SiComPre 1.0 to predict protein complexes. **b** An example of how Markov Clustering (MCL) can split bounded protein complexes. **c** Example of how the merging step combines two highly similar simulated complexes into a refined complex

Like in our previous method,^1^ the SiComPre 1.0 simulate stochastic complexation, decomplexation and diffusion of proteins in a 2D space, dynamically generating a list of complexes (called Simulated Complexes, SCs), which show quite robust distribution of the various complexes, independent of initial condition and simulated time (after a transient period). To obtain both a qualitative and quantitative prediction, the software refines the prediction in order to generate a list of refined complexes (RCs) together with their abundances (Fig. 1a). SiComPre 1.0 first splits complexes where single edges link strongly connected subnetworks by applying Markov Clustering^9^ on the network representing protein complex structures generated from the simulation. We observed that with a low threshold we can split protein complexes that are connected only through a few interactions (Fig. 1b), which might be art-facts due to the lack of data on sterical inhibition of binding events on some molecules involved in multiple complexes. Next, similar SCs that differ only in a few proteins are merged together (Fig. 1c). For this purpose, we built a similarity matrix where each element represents the overlap score between two complexes, then a hierarchical clustering can be applied to the matrix and an iterative process to merge pairs of complexes starting from the most similar ones (Supplementary Text 1). The process stops when there are no couples of complexes with an overlap score greater than the default similarity score (overlap > 0.5) (Supplementary Text 1). The result is a list of RCs that serve as qualitative predictions of protein complexes. These can be compared to lists of experimentally identified real protein complexes.^10–15^ Afterwards, SiComPre assigns SCs, obtained after the splitting step, to the RC with the highest overlapping score. The amount of SCs matching an RC constitute defines the quantitative prediction for each protein complex. We note that this process does not always merge all the simulated protein complexes that correspond to the same real complex to a single RC. Even with this approach we find some RCs that cannot be merged because their similarity is below the threshold, but both of their similarities to a common real protein complex are above the cutoff. These RCs can be considered as alternative forms of the same protein complex and their respective quantities (number of SCs assigned to them) could give a prediction of the distribution of the various alternative forms.

### User-friendly implementation

The implementation of the above described pipeline and additional features to elaborate and visualize the results are accessible through a graphical user interface. More specifically, users can import data on PPI, DDI, GO Functions and various other protein attributes (diffusion rate, abundance, localization and available binding surfaces) as well as select parameters for model generation and simulation (Supplementary Text 1). Users can draw their custom compartmentalized cell or select a default compartmental model to define the spatial simulation environment, they can also decide the number of simulation threads and if to run a hybrid computation (GPU + CPU) or CPU-only computation. For proteome-wide simulations we recommend the use of hybrid computation. Additionally, we provide scripts to convert some of the most common database formats^8,16,17^ into a format directly used as input for SiComPre 1.0 (Supplementary Text 1). Windows and Linux compatible versions of SiComPre tool can be downloaded from https://bitbucket.org/sicompre/sicompre or https://www.cosbi.eu/research/prototypes/sicompre. Hardware requirements for toy models are minimal, but for proteome-wide simulations a machine with large memory and GPU acceleration is preferred. Details on software preformances are presented in the Supplementary Text 1, where we also explain how example simulations can be performed.

Once the simulation is finished the graphical user interface enables the user to track and visualize the diffusion of a selected list of proteins during the simulation. It is also possible to export information about the predicted complexes (their abundances, list of constitutive proteins, and a GO enrichment on them) and the score of the given predicted complex against a reference database, with the best-matching known complex.

We also added a functionality to automatically compare results of multiple sets of simulations. This process identifies clusters of structurally similar complexes across multiple simulation results (e.g. generated from different inputs) and report variations in their abundances (Supplementary Text 1). These results can be used to evaluate qualitative and quantitative differences between protein complexes predicted from models generated from different input data (e.g. protein abundances retrieved from data on liver against protein abundances in pancreas).

Another key feature of SiComPre 1.0 is the possibility to analyze the specificity of proteins constituting protein complexes. Users can export a list of protein complexes, together with the abundance of each protein within a given complex and their total abundance. The combination of these abundances gives a specificity measure (the fraction of proteins in the whole cell that are found in simulated complexes matching the selected RC) of each protein to each protein complex, which can be used to design the best baits for any protein complex (Supplementary Figure S1). Using the option “export to network” (in a format that can be imported into Cystoscape^18^), users can generate networks of the predicted protein complexes containing info on the abundance of proteins participating in a given complex (how many times a protein appears in a simulated complex that has the selected RC as best matching), with some proteins appearing in multiple copies in a given complex. The exported file also gives information on the specificity of proteins in a complex as defined above.

## Results

We assessed the new features introduced in SiComPre 1.0 to predict the characterized complexome of *Saccharomyces cerevisiae* to measure the qualitative prediction performance of SiComPre 1.0 against a large set of validated data. To highlight the unique features of SiComPre 1.0, we also investigated the qualitative and quantitative variation in the complexome of the mouse liver before and after Metformin treatment. Finally, we ran SiComPre 1.0 with input data on tissue-specific protein abundances of human cells^19^ to investigate variations in the complexome between tissue types.

In all the experiments, predicted complexes were compared to a reference dataset using the composite score of recall, maximum matching ratio (MMR) and accuracy.^20^ Yeast complexes were compared against the CYC08 dataset,^21^ human and mouse results were compared to the CORUM dataset.^22^ These predictions against a reference dataset can be replicated and extended by users of SiComPre 1.0.

### Test case 1: Yeast complexome

As an example, SiComPre 1.0 was tested with the following input data on yeast: PPIs from Collins et al.^23^ protein localization and molecular function from MIPS^24^ and protein abundances from Ghaemmaghami et al.^25^ Protein domains association are retrieved from SMART^26^ and IDDI^27^ was used as DDI dataset. We first tested new features of SiComPre 1.0 on the limit of the total possible binding to each protein and the effects of incorporating compartmental localization data on each protein (Supplementary Text 1). We considered each predicted complex as a match to a real complex (based on CYC08^21^) when there was an overlap score >0.25. According to the optimal composite score values,^28^ we found that 4096 subvolumes and a binding limit of 10 simultaneous partners give the best results (Supplementary Text 1 and Supplementary Table S1). Of course, some scaffolding proteins could have more binding partners, while small proteins could have less. This is the optimized value what we could use for all in a proteome-wide simulation, but the input files of SiComPre 1.0 enable users to define this limit for each individual protein.

**Fig. 2:**
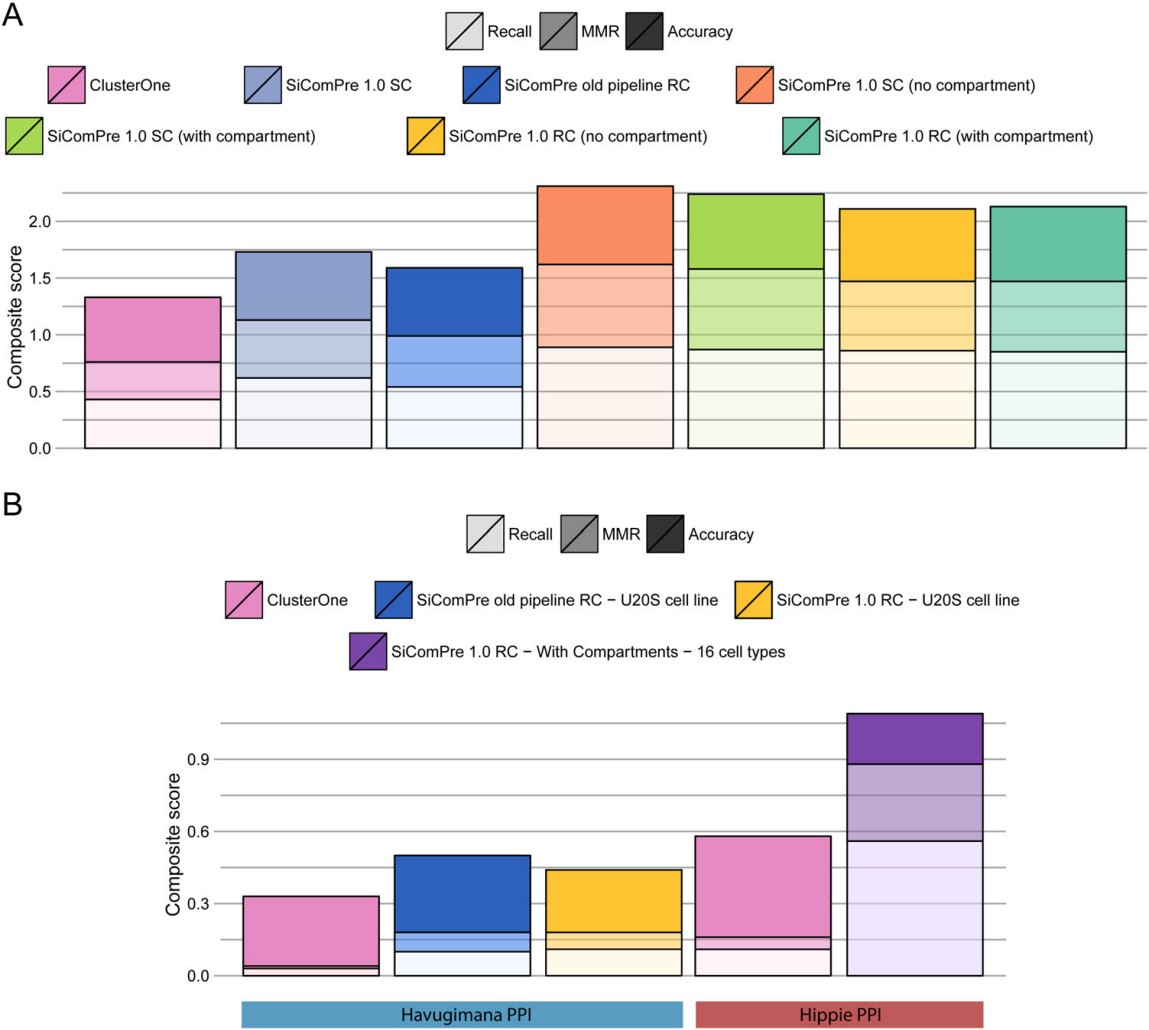
Composite score of protein complex prediction in yeast and human cells. **a** For yeast, both SiComPre versions and ClusterOne^20^ predictions were evaluated, based on the CYC08 reference dataset^21^ and Collins PPI^23^ as an input. **b** For human data we tested the tools on two different PPIs (Havigiumana and Hippie),^40,54^ with the CORUM as a reference dataset.^22^ Abundances for SiComPre models were retrieved from U2OS cell line^55^ and various other tissues and cell types.^19^ Additional combinations of used input datasets are available in Supplementary Figures S2 and S4

Next, we ran SiComPre 1.0, with and without compartments, with the above identified settings. Without using data on compartmental localizations, SiComPre 1.0 achieves a better composite score (0.1 higher) than with data on compartments (Supplementary Text 1 and Supplementary Table 2). However, an important consideration to predict protein complexes is to generate a prediction that is consistent with the localization of proteins. For this, we evaluated our prediction after filtering out complexes which contained proteins that has been previously shown to be localized in separate compartments.^8^ Following filtration SiComPre 1.0 with compartmental information provided a better prediction (Fig. 2a). A detailed comparison of SiComPre against other existing methods was previously performed.^1^ Here, we compared the location-consistent protein complexes with the predictions obtained from SiComPre 1.0, SiComPre with the same parameters as defined in the first version of the method^1^ and ClusterOne.^20^ The latter is a successful clustering algorithm to detect overlapping communities in PPI networks. As opposed to SiComPre, ClusterOne only needs a PPI dataset as input; however, it cannot provide quantitative prediction of protein complexes and it does not consider protein localization. For this analysis we also filtered out reference complexes that were not in the original PPI,^23^ to ensure that we measure the quality of the method rather than the quality of the PPI input data. SiComPre 1.0 outperforms both ClusterOne and the older SiComPre pipeline (Fig. 2a and Supplementary Figure S2A). Interesting to note that the performance scores are reduced with the consideration of compartmental localization of proteins. This is due to the fact that reference protein complex databases contain a large number of complexes (79 in yeast, 131 in mouse and 227 in human) that are composed of proteins that are not localized at the same compartment, based on various localization resources.^8,24^

In the generated SiComPre 1.0 model, we observed that SiComPre 1.0 added at least one fictitious domain (i.e. there is no information on known interacting domains associated to the interacting proteins) to 1036 proteins. As expected, a large portion (*n* = 360) of them were associated to ribosomal proteins in GO, suggesting that ribosomal proteins do not interact with each other directly, rather they interact through ribosomal RNAs (rRNAs). Therefore, we added protein−rRNA binding information^29^ to the simulation (Supplementary Text S1). This further increased the composite score achieved by SiComPre 1.0 to 2.15, from 2.13 obtained without rRNAs and their interactions (Supplementary Figure S2A).

With SiComPre 1.0 it is possible to predict the abundance of protein complexes in a cell by merging similar SCs into RCs and count the overlapping SCs for each RC. These results can be exported as CSV file together with other features (e.g. list of constitutive proteins, complex size, GO enrichment). Separately, we validated our quantitative predictions against the limited protein complex abundance data from the literature.^10–15^ We found quantitative data on the abundance of nine protein complexes; SiComPre 1.0 predictions show a Pearson’s correlation of 0.77 with a *p* value of 0.015, against this data (Supplementary Table S3). SiComPre 1.0 also outperforms the trivial method of predicting protein abundances by averaging the abundance of the proteins in the reference complex, which did not significantly improve the random prediction obtaining a Pearson’s correlation of 0.28 with a *p* value of 0.47 (Supplementary Table S3), and earlier SiComPre prediction (Pearson’s correlation = 0.41, *p* value 0.31).^1^

Since there are limited data on protein abundances after various perturbations, it is often desired to use gene expression data as a proxy for protein abundances. To assess SiComPre 1.0 prediction with gene expression instead of protein expression, we tested SiComPre 1.0 using mRNA abundances instead of proteins.^30^ Although expression of many proteins is post-transcriptionally regulated, this is helpful to extend the applicability of SiComPre 1.0 to a larger spectrum of conditions. Nowadays gene expression quantification is available for multiple cell types and organisms obtained from different experimental conditions and also at the single-cell level.^31^ Furthermore, these data have typically a larger coverage compared to proteomics data. This method of using mRNA abundances instead of protein abundances led to a decrement in the qualitative matching composite score to 2.0, but at the same time the quantitative predictions increase to a Pearson’s correlation of 0.94 (Supplementary Table S3). This result suggests that when proteomics data are unavailable then mRNA abundances can also be used as a reliable input of SiComPre 1.0, since the qualitative scores are still reasonable. The quantitative prediction seems to improve with mRNA data used, but this was evaluated based on a limited set of complexes with known abundances, most of which are essential (e.g. ribosomes). These complexes might be less controlled on the post–transcriptional level, so this good quantitative prediction might not extend to other protein complexes under various controls.

To test how well SiComPre 1.0 can be used to predict the best baits to pull down each protein complex in yeast, we calculated the specificity, abundance, and degree of proteins within each complex and compared these with the list of baits used in Gavin’s experiment.^32^ The ROC curves of the three measures show that the specificity and to some extent the abundance of proteins in individual protein complexes can be used as best predictors of good baits (Supplementary Figure S3). These measures can help to design coimmunoprecipitation or tandem affinity purification experiments by identifying highly specific proteins in the complexes. Furthermore, the predicted baits could also be used as drug targets to interfere with the activity of each protein complex. To further emphasize the robustness of the simulation, it is important to note that we got these results without the need of training our algorithm as usually done for classifiers; we just relied simply on SiComPre 1.0 simulation results.

The whole in silico experiment on the yeast proteome can be performed in 1 day on a regular PC with a GPU (core i7 and NVidia Geforce GTX 760M), with the reduction of protein abundances to the square root of measured values.^25^ Use of real protein abundances can be considered only on high performance computing clusters. In the supplementary information, we show how the performance of SiComPre 1.0 depends on the actual protein abundances (Supplementary Text 1 and Supplementary Table S4). A good qualitative fit can be achieved even with greatly reduced abundance values at a much shorter computing time. A step-by-step protocol to execute the pipeline is explained in Supplementary Text 1.

### Test case 2: Metformin in mouse liver

**Fig. 3:**
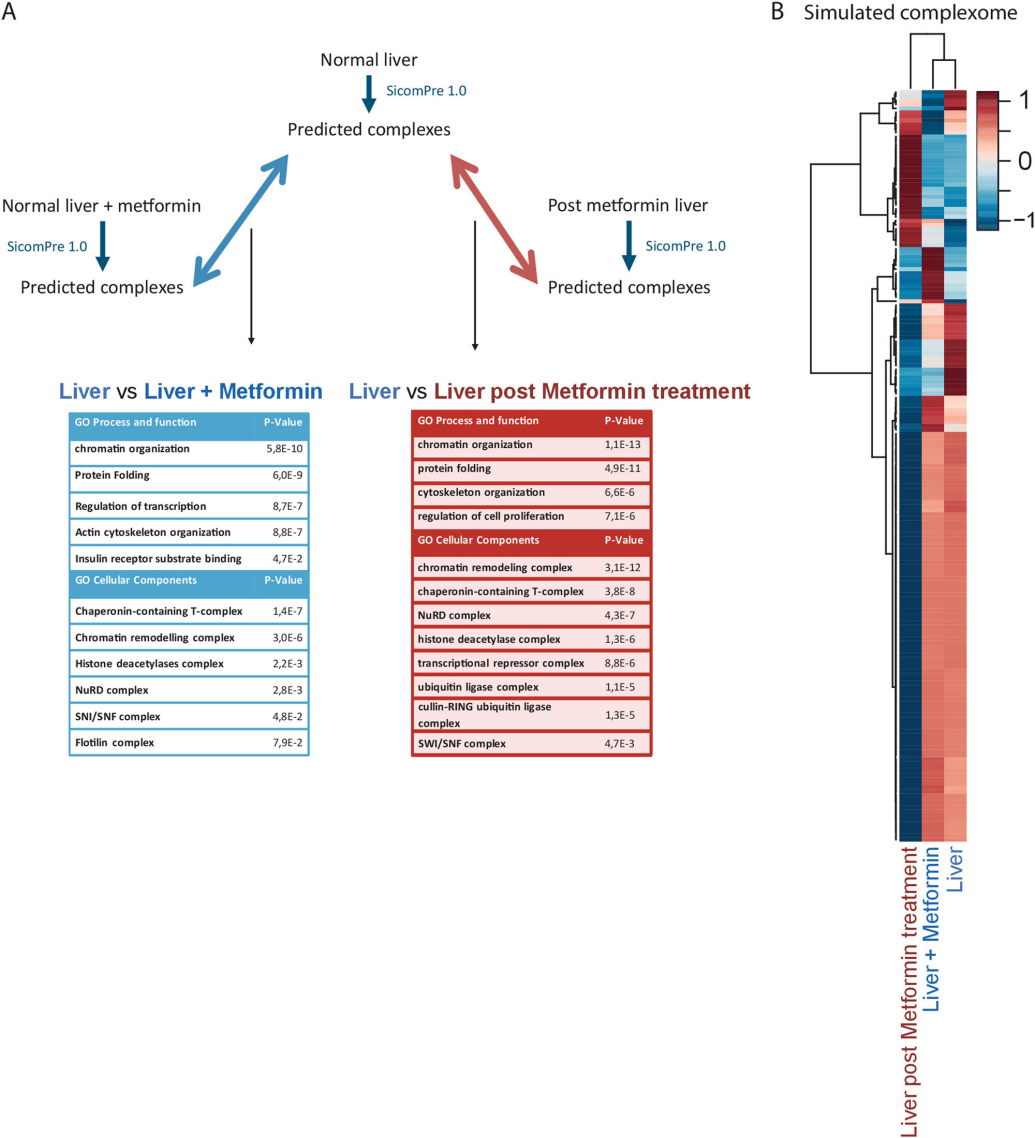
Protein complex variations in mouse liver after Metformin treatment. **a** Complexome comparison between in vivo and in silico addition of metformin and GO terms enriched in the most varied protein complexes. **b** Heatmap of protein complex variability between the three conditions. Hierarchical clustering is based on the Euclidian distance between normalized protein complex abundances. Color scale represents the estimated complex abundance, where red are the highest and blue the lowest abundance complexes

Metformin is a drug used to treat type II diabetes, but its molecular mechanism is still unclear. It does not affect insulin production in the pancreas, but is more likely to reduce hyperglycemia, by activation of AMP-activated protein kinase (AMPK). The primary action, according to Drugbank,^33^ is to induce prkaa1, scaffold protein for the formation of AMPK complex, whose functions are to regulate cellular energy, metabolism and it acts as a regulator of cell polarity by remodeling actin cytoskeleton. To exploit the potential of SiComPre 1.0 in predicting variation in the complexome upon perturbation, we compare protein complexes predicted from protein abundance data on mouse liver before and after metformin treatment. SiComPre 1.0 cannot model post-translational modifications, but it can give an insight about which protein complexes might be affected upon perturbation by introducing a new molecule into the system. We ran a set of three simulations with a total of six timepoints for each of the following protein abundance setups: (i) normal liver protein abundances, (ii) post metformin treatment liver protein abundances and (iii) normal liver protein abundances, plus metformin with its molecular bindings, retrieved from STITCH.^34^ We compared these simulations in two different ways to test how well SiComPre can predict the effect of metformin on the liver complexome. We compared the simulated complexome of the normal liver (i) to the experimentally perturbed system (without adding metformin to the simulations, but considering abundance changes as an effect of metformin), and (ii) to the system, where in silico we added only metformin with its interactions to the normal complexome. Protein abundances data came from MOPED,^17^ PPI from Biorgrid,^35^ DDI from IDDI,^27^ protein domain from SMART,^26^ protein localization and protein function from GO.^8^ We used different input resources to highlight that SiComPre 1.0 can handle various input formats. In the supplement we provide a series of scripts that can help users to transform downloaded data from various online resources into a format readable by SiComPre 1.0 (Supplementary Text 1). For the in silico addition of the drug we integrated protein−drug interaction from STITCH^34^ with protein abundances retrieved before drug administration. Protein binding sites interacting with Metformin were selected performing a domain enrichment with DAVID^36^ on the list of proteins interacting with Metformin. Using the option “compare conditions” of SiComPre 1.0, we generated a table reporting all the protein complex abundances and structural differences detected between the different sets of simulations. Next, we ranked protein complex variations according to their coefficient of variation between the two compared conditions. In Fig. 3a we list the GO terms enriched for protein complexes with high variation between compared experiments.

Heatmap of protein complex abundances show that the experimental metformin treatment caused a large effect on the abundance of proteins and as a result on the abundance of protein complexes (Fig. 3b), while the in silico addition of metformin had a much smaller effect. In the experimental case this could reflect the downstream effects of the treatment, while in the in silico, this might show the direct effects of metformin on the complexome. The altered proteins of the complexes in both cases were enriched in protein folding processes and chromatin and cytoskeleton. This inhibition of unfolded protein response by metformin has been already reported.^37^ In addition, the observed relationship between metformin and histone levels have been identified earlier.^38^ Furthermore, SiComPre 1.0 also suggests that metformin affects cell motility and the cytoskeleton, as shown in PC3 and DU145 prostate cancer cells.^39^ The predicted complexes responsible for this overlapping pattern can be matched to the chaperonin containing TCP1 complex, p97-Ufd1-Npl4-IP3 receptor complex, Smcb-Smcd-PW29 complex, Xin-Cdh2-Ctnnb1-Ctnnd1 complex, and Kif3-cadherin-catenin complex.

### Test case 3: predicting the tissue-specific human complexome

The new SiComPre pipeline is designed to increase the performance on the human proteome and allow prediction of tissue-specific protein complexes. Therefore, after assessing the quality of our prediction in yeast and showing how SiComPre 1.0 responds to perturbations such as addition of new molecules, we applied our method to identify how complexes vary among seven different adult tissues, six fetal tissues, and four different primary hematopoietic cell types. The protein abundances in each of these cell types were recently measured.^19^ Multiple PPI datasets were tested and we found that the Human Integrated Protein−Protein Interaction Reference, called Hippie^40^ was performing better than any other dataset with both SiComPre 1.0 and ClusterOne (Supplementary Text 1 and Supplementary Table S5**)**. Using this dataset as the PPI input dataset, SiComPre 1.0 was able to identify a total of 56% of all the complexes collected in the CORUM reference dataset.^22^ The tissue with the highest percentage of matched complexes was the adult testis (29%), while the data from adult heart tissue gave the lowest matching (15%) to protein complexes in CORUM. In comparison, ClusterOne^20^ could predict only 10% of these complexes, highlighting that SiComPre 1.0 and the incorporation of protein abundance data could help better predictions (Fig. 2b). Tests on another PPI dataset led to lower prediction scores for all methods, but SiComPre 1.0 still had the best performance of all investigated software (Fig. 2b and Supplementary Figure S2B). We also compared SiComPre 1.0 with other existing methods^9,20,41–44^ on the datasets from 16 cell types and SiComPre gave a far higher *recall* and an elevated *MMR* compared to any other methods leading to SiComPre reaching the highest *composite score* (Supplementary Figure S4). Interestingly, the average Pearson’s correlation between the abundances of predicted complexes across different cell types is 0.68, compared to 0.58 calculated directly from protein abundance correlations. This suggests that complex formation might have a role in reducing the heterogeneity caused by differences in protein expression levels.

We could use again the predicted complexome comparison feature of SiComPre 1.0 to investigate alteration in protein complex abundances in different cell types. By ranking protein complexes according to the coefficient of variations of their abundances among different cell types, we identified desmosomal cadherin-plakoglobin complex, proteasome and spliceosome as the most highly variable complexes. It was already reported that proteasome can perform tissue-specific functions;^45^ thus it was interesting to notice that the subunit of the proteasome involved in transcriptional regulation (GO:0008134, GO:0003713) and DNA binding (GO:0003677) was more abundant in B cells, frontal lobe and adult monocytes, while the subunit involved in threonine-type endopeptidase and peptidase activity (GO:0004298) was more abundant in T_H_ cells, testis, and adult ovary (Supplementary Figure S5). Spliceosome also has been reported as highly variable^46^ and we indeed found a complex partially matching the spliceosome (12 proteins out of 16 belong to the spliceosome) as the complex with the highest variability in its abundance. It was highly abundant in adult B and TH cells and in fetal ovarian cells. To investigate how the complexome of different cell types differ from each other we performed a principal component analysis based on SiComPre 1.0-predicted complex abundances (Supplementary Figure S6). We were able to separate cells of the reproductive organs through the first principal component, immune system cells using the second principal component and adult from fetal tissues with the third principal component. The most influential complex of the first principal component was CKB + ASB9 complex,^47^ for the second one the most important complex was hemoglobin and for the third CST3 + C4A complex. As a control, we repeated the same analysis generating a PCA with original protein abundances^19^ as input. This way, it was possible to separate reproductive organ and immune system cells, but adult and fetal cells were mixed together (Supplementary Figure S7). In another control, we considered only complexes defined in CORUM database^22^ and we took the average of the protein abundances found in a given complex as predicted protein complex abundance. This analysis did not find any clustering that enabled separation cell types (Supplementary Figure S8). As a further validation, we investigated whether SiComPre 1.0 was able to predict complexes that are not part of the reference CORUM database. Among these SiComPre prediction we found multiple variants of the SET/MLL complex family.^48^ SiComPre predicted three variants of this complex, matching Set1A, Set1B, and MLL protein complexes (Supplementary Figure S9A). Furthermore, our quantitative prediction identified Set1A as the most abundant proteins in its respective complex, highlighting the importance of having a quantitative prediction of protein complex. ClusterOne,^20^ the tool with the highest accuracy measure (Supplementary Figure S4), was not able to distinguish between these highly similar complexes predicting only one complex that partially match all SET variants (Supplementary Figure S9B). This piece of result highlights one of the special features of SiComPre, that it is capable of distinguishing complexes with high similarity.

## Discussion

Here we presented SiComPre 1.0, a user-friendly and cross-platform software tool that enables qualitative and quantitative predictions of protein complexes from data on protein−protein interactions, protein abundances, and protein domains. Compared to our earlier released method,^1^ the updated SiComPre can be used to investigate context-dependent changes in the complexome. The tool now allows the comparison of simulation results from input data from two separate datasets (e.g.: before and after treatment by drug (Fig. 3), between data from various tissue types). It also allows direct comparison with data on known protein complexes. Users can define compartments and the simulation space uses this data to allow complex formation inside a single compartment or between molecules present in adjacent compartment (e.g.: cytoplasmic and ER proteins). We have changed the complex refinement algorithm (Fig. 1), which leads to much better performance (Fig. 2). We provide scripts to convert output files of major databases into the form they could be loaded into SiComPre. The simulations can now be performed also on CPUs, not only on GPUs as before. Simulation parameters can be manually tuned (number of runs, conversion factor, use of GO in prediction); users can also define diffusion rates and maximum binding partner numbers in the input files. These all are easily accessible in a user-friendly graphical interface. Such software tool is currently missing from most other protein complex prediction methods, making SiComPre not only one of the best performing tools, but also the most accessible.

We demonstrated the workflow of the pipeline using three test cases and explained how the results of SiComPre 1.0 analyses were validated with independent experimental results (Fig. 2, Supplementary Figure S3, and Supplementary Table S3). By incorporating data on compartmental localization of proteins, we improved predictions for the human protein complexome, almost doubling the number of successfully predicted complexes compared to earlier work (Fig. 2b). To avoid overfitting, we restricted our earlier pipeline to only biologically important steps that were not aimed to improve the score. Our predictions based on data on protein abundances before and after metformin treatment^17^ allowed us to identify variations in protein complexes related to unfolded protein response, which could not have been reported by methods solely based on protein abundances.

There are many other potential utilizations of SiComPre 1.0. It is possible to use the features of SiComPre 1.0 to get information about protein complex compositions (e.g. network export) that can be used to drive proteomic experiments and to reveal new drug targets. To investigate competing binding events, the tool allows users to investigate what complexes are formed in a limited set of proteins and it also can predict the quantities of the competing complexes. SiComPre 1.0 can be also used to test changes in the complexome for perturbations where protein abundances change within cells (siRNA, gene deletion, overexpression, etc.).

The accuracy of qualitative and quantitative predictions is interdependent. We cannot have a good quantitative prediction with a wrong qualitative prediction (Fig. 2b). Furthermore, we cannot predict good protein complex markers without a good quantitative prediction. This is because in SiComPre 1.0 predictions the specificity, abundance, and degree of the proteins belonging to a complex are based on the qualitative and quantitative structure of the predicted complex.

With the advent of new experimental techniques, novel context-specific data are becoming available. These provide an exciting opportunity for SiComPre 1.0 users to study how protein complexes vary in different conditions. For instance, Schmidt et al. recently quantified the absolute protein abundance in *Escherichia coli* cells under 22 different growth conditions^49^ and data generated on further tissue-dependent expressions in multiple organisms.^50,51^ It was also shown how to quantify proteins at the single-cell level.^52^ These data can be translated into single-cell complexome prediction with SiComPre 1.0 to explore cell-to-cell variability in terms of protein complexes. In general, SiComPre 1.0 allows users to quickly analyze quantitative proteomics data and predict differences in the complexome. This could lead to useful insights to design further functional experiments.

SiComPre 1.0 is still not able to dynamically capture the expression and degradation of proteins over time as this will require a more computationally intensive simulation, making the analysis of human data nearly impossible. However, users with time-dependent data can use such data as input of SiComPre 1.0 to obtain protein complex predictions for multiple time points and compare how the complexome changes in time. Currently, predictions are based on approximated, fixed parameters for all proteins (binding, unbinding rates and diffusion constants), but as these become available the software already allows to use individually measured rates for each protein and interaction.

## Materials and methods

### Metrics for protein complex evaluation

Predicted protein complexes were compared to a reference dataset.^21^ We considered a successful prediction on protein complexes with an overlap >0.25, where the overlap score for a predicted complex *p* compared to a reference complex *r* is defined as follows:

$${\mathrm {Overlap}}\left( {p,r} \right) = \frac{{|p\mathop { \cap }\nolimits r|^2}}{{|p| \times |r|}}.$$

A combination of different measurement scores has been utilized to assess SiComPre performance against other tools.

*Recall***:** the fraction of successfully predicted protein complexes in the reference dataset.

$${\mathrm {Recall}} = \frac{{\left| {\left\{ {b{\mathrm{|}}b \in B,\exists p \in P,Overlap\left( {p,b} \right) > \omega } \right\},} \right|}}{{\left| B \right|}},$$

*ω* is the threshold value, *P* is the set of simulated complexes and *B* of reference complexes.

*Sensitivity*: the fraction of proteins in reference protein complex *i* which are found in a predicted complex *j*, ${\mathrm {Sn}}_{i,j} = T_{i,j}/N_i$, $T_{i,j} = Vi \cap Vj$ and $N_i = \left| {Vi} \right|$. While, *the real complex-wise sensitivity* is the maximal fraction of proteins of complex *i* by its best-matching simulated complex $n_{co_i} = \max\nolimits_{j = 1}^m{\mathrm {Sn}}_{i,j}$. Finally, the general sensitivity or *complex-wise sensitivity* is the weighted average of *real complex-wise sensitivity* over all complexes

$${\mathrm {Sn}} = \frac{{\mathop {\sum }\nolimits_{i = 1}^n N_i{\mathrm {Sn}}_{co_i}}}{{\mathop {\sum }\nolimits_{i = 1}^n N_i}}.$$

*Positive predictive value*: the number of proteins in a predicted complex *j* that belong to a real complex *i* over the total number of proteins of predicted complex *j* assigned to all complexes, ${\mathrm {PPV}}_{i,j} = T_{i,j}/\mathop {\sum }\nolimits_{i = 1}^n T_{i,j}$. As above there is also a complex-wise predictive value, ${\mathrm {PPV}}_{cl_j} = \max\nolimits_{i = 1}^n{\mathrm {PPV}}_{i,j}$. While the general PPV is

$${\mathrm {Sn}} = \frac{{\mathop {\sum }\nolimits_{j = 1}^m T_{.j}{\mathrm {PPV}}_{cl_j}}}{{\mathop {\sum }\nolimits_{j = 1}^m T_{.j}}}$$

where $$T_{.j} = \mathop {\sum }\nolimits_{i = 1}^n T_{i,j}$$.

*Accuracy*: geometric accuracy is the geometrical mean of Sn and PPV, ${\mathrm {Accuracy}} = \sqrt {{\mathrm {Sn}} \times {\mathrm {PPV}}}$

*MMR*: is a measure proposed by Nepusz et al.^20^ which tries to find the better correspondence between simulated complexes and reference complexes. This value can be calculated as maximum bipartite matching in weighted graph, where nodes corresponding to a predicted complex are connected with an edge to a node representing a real complex where its weight is the overlap value between the complexes.

### Simulations

All the simulations have been performed with SiComPre 1.0 GUI. For all simulations grid dimension was 64 × 64 SVs and SiComPre was executed with default parameters. Only for human and mouse simulations, cell volume was set to 1000, because protein abundances were expressed in molecules/million. For yeast simulation, maximum concentration of protein in one SV was set to 150, while in human the value used was 1500. Size of compartments has been chosen according to the square root of the volume reported for liver cells.^53^ Next, small adjustments were adopted to better fit the size to the number of proteins in the relative compartment. The input data used for the experiment is located and described in the “Data” directory of SiComPre 1.0.

### Definition of specificity of a protein in a given protein complex

The specificity for a protein *i* in a complex *c* is defined as $S_i^c = A_i^c/A_i$, where $A_i^c$ denotes the abundance of protein *i* in a complex *c*, while *A*_*i*_ is the total abundance of the protein *i* in the system.

### Statistical analysis

Statistical analyses have been performed in R and Python. Function enrichment analysis was performed with DAVID.^36^

## Electronic supplementary material

Supplementary Text 1

## Data Availability

All data used for simulations were collected from public resources, the software is freely downloadable from https://bitbucket.org/sicompre/sicompre or https://www.cosbi.eu/research/prototypes/sicompre.

## Electronic supplementary material

**Supplementary information** accompanies the paper on the *npj Systems Biology and Applications* website (10.1038/s41540-018-0073-0).
