# Supplementary material for: Context-dependent prediction of protein complexes by SiComPre
Source: NPJ Syst Biol Appl. 2018 Sep 17;4:37. doi: 10.1038/s41540-018-0073-0 (PMC6141528; doi:10.1038/s41540-018-0073-0)
Supplement: Supplementary file 1 — Supplementary Text 1 [file 41540_2018_73_MOESM1_ESM.pdf]

Supplementary Text 1 for
“Context Dependent Prediction of Protein Complexes by SiComPre”

Simone Rizzetto[1,2], Petros Moyseos[1], Bianca Baldacci[1], Corrado Priami[1,3], Attila Csikász-Nagy[4,5]

1. The Microsoft Research – University of Trento Centre for Computational and Systems Biology (COSBI), Piazza Manifattura, 1, 38068 Rovereto (TN), Italy
2. School of Medical Sciences, UNSW Australia
3. University of Pisa, Department of Computer Science, Italy
4. Randall Centre for Cell and Molecular Biophysics, King's College London, London, UK
5. Faculty of Information Technology and Bionics, Pázmány Péter Catholic University, Budapest, Hungary

Contents

1. Information on the pipeline of SiComPre 1.0 2
 1.1 Implementation 2
 1.2 Compartment definition and simulation 2
 1.3 Controllable parameters 3
 1.4 Parameter tuning 4
 1.5 Algorithm to merge similar complexes and reach Refined Complexes 6
 1.6 Compare simulation results from different conditions 8
2. Additional results 8
 2.1 Performance with reduced protein abundances 8
3. Step-by-step tutorial to execute SiComPre 1.0 9
 3.1 Step 1. Requirements 9
 3.2 Step 2. Define compartments 10
 3.3 Step 3. Import input files and generate the model 11
 3.4 Step 4. Run simulations 13
 3.5 Step 5. Refine complexes 15
 3.6 Step 6. Check and export results 16
 3.7. Save and Load sessions 18
 3.8. Execute SiComPre from the command line 18
 3.9. Run a test 19
 3.10. Utils provided as separate scripts 19
4. Selection of the best PPI for human studies 20
5. Additional steps for reproducibility 22

5.1. Protein complexes localization 22
5.2 Estimate protein-rRNA interactions and rRNA abundances 22
6. References: 23
7. Supplementary Figures and Tables 27

1. Information on the pipeline of SiComPre 1.0

1.1 Implementation

The simulation algorithm has been implemented in C and CUDA for GPU computation. All the remaining scripts as well the GUI have been implemented in Python 2.7 using numpy, matplotlib, pubsub and wx modules. Executable of the GUI have been generated with PyInstaller. We also provide a python script to execute the whole pipeline from command line. This is particularly useful for users who can access high performance computing facilities.

SiComPre 1.0 is available both for Windows and Linux operating systems.

Execution of this pipeline with one simulation only on a desktop with intel core i7, GeForce GTX 760 and 16 GBs RAM takes proximally 4 hours for models generated from yeast proteome and 24 hours from human proteome data. The example provided within SiComPre 1.0 (Supplementary Text 1 and Supplementary Table 6) can be completed without GPU in few minutes.

1.2 Compartment definition and simulation

Compartments can be defined by users, but we still provide a default compartment organization. Protein assigned to a compartment can only diffuse in SVs that are part of the compartment. In our default model, we assign a group of SVs to each compartment. It is also possible to assign more compartments to a single SV in order to generate an overlap where proteins of different compartment can meet. This is useful to predict transmembrane protein complexes. From a simulation point of view proteins can interact with every protein in the same SV, however in the model used as example we didn't generate an interaction for proteins that are not in the same

compartment except if the compartment is a membrane. As an example, cytoplasm and Golgi are in contact and they share some of the assigned SVs. A third compartment is used to define the membrane between Golgi and Cytoplasm. Proteins in the cytoplasm can reach the membrane as well those in the Golgi. However, they cannot interact because in our model we didn't allow the interaction between two compartments. Instead, proteins in the cytoplasm and Golgi can interact with proteins in the membrane. Thus, through membrane protein we can model transmembrane protein complexes. Size of compartments has been chosen according the square root of the volume reported for liver cells [1]. Next, small adjustments were adopted to better fit the size to the number of proteins in the relative compartment.

1.3 Controllable parameters

The simulation results are dependent on multiple parameters that can be tweaked by users according their needs. Below we summarize the parameter functionalities divided by their stage in SiComPre 1.0 pipeline:

- Compartments:
    - Grid dimensions: the number of sub-volumes (SVs) used to discretize the simulation space.
- Model generation:
    - Binding strategy: the strategy used to resolve ambiguity in decomposing PPIs into DDIs. Possible values are Fictitious, Non Fictitious, and Functions.
    - Cell volume: protein abundances given in the input file are multiplied by this number.
    - Abundance reduction: is a value between 0 and 1; protein abundances after cell volume multiplication are raised to the power of this number.
- Simulation:
    - Maximum concentration: number of proteins that a single sub-volume can simultaneously contain.
    - Simulations: how many times a model will be simulated.

- Maximum simulation time: if defined, SiComPre stops when the simulation time reaches the indicated value.

- Refine complexes:
  - Merging threshold: threshold to merge similar complexes when the refined list of complexes is generated.

1.4 Parameter tuning

We tested SiComPre 1.0 on data from the yeast *Saccharomyces cerevisiae* with various assumptions:

- We tried setting the protein binding limit (BL) to 10 or to 100, meaning that in one case the maximal number of binding partners of each protein was assumed to be 10 or 100 (quasi no limit). This was done to see whether this feature has an impact on the prediction of protein complexes.
- To see if there is any difference in the predicted complexes varying the number of SVs used in the simulation we run the simulations multiple times with SV=4096 and SV=1024.
- We also tested if restrictions on the compartmental localization of each protein has an effect on the results, thus we ran simulations with and without considering compartments.
- We set the number of simulations to 3 for each combination of parameters and as there is no proteome-wide information available on it, we set the binding and unbinding rates to 1.

Predicted complexes were compared to a reference dataset [2] using the composite score of recall, maximum matching ratio (MMR) and accuracy [3] and removing complexes whose proteins were not found in the initial PPI, in order to assess the method rather than the initial PPI dataset. We tested our simulations with multiple parameter choices (**Supplementary Table 1**).

Interestingly the consideration of compartments reduced the matching scores on the simulated complexes (SCs). Setting the binding limit of each protein to 10 increased the composite score, compared to BL=100 (minimal steric restriction on binding partners). Simulations with 1024 SVs reduced the composite score compared to SV = 4096. All of these scores were calculated

removing complexes whose proteins were not found in the initial PPI, in order to assess the method rather than the initial PPI dataset. According to these scores, we selected as best option SV=4096 and BL=10, because with the same composite score it has a higher precision (0.4648 vs 0.4576) when compartments are not considered and it has a higher composite score with compartments. Note that we didn’t run the whole pipeline for each combination, since our purpose was to see how the simulation performs with each parameter.

Next, we ran our simulated complex refinement pipeline with different settings and finally we checked the score of complexes enriched in the same compartment (**Supplementary Table 2**). Without compartments SiComPre 1.0 can get a better composite score, however the resulting complexes are not consistent with compartments. Including the split step, the score improves by 0.5 without using compartments and 0.2 using compartments. The maximum composite score is reached with the splitting algorithm, but without compartments (2.38). In order to assess the method rather than the initial PPI dataset, all scores were calculated removing complexes whose constituents were missing from the initial PPI dataset.

Split is based on Markov clustering (MCL) with the inflation parameter equal 1.4. We observed that with this parameter MCL allows to split complexes without breaking single subunits and we can split protein complexes that are bounded by a weak interaction (Fig 1B). Next, we merged complexes according to their similarity (Overlap score > 0.5). These two steps can be performed once the simulations are over. Although the process of merging similar complexes decreases the overall composite score, it is a necessary step to reduce the noise in our qualitative prediction and to have a reliable quantitative prediction, because many multicomponent complexes has a large number of variants, but in the predictions these should be considered the same complex instead of many different low abundant predictions. Users with interest in all the alternative forms can stop the pipeline at the simulation step and investigate those results.

As suggested by Nepusz et al. [3], due to the lack of completeness of reference dataset of protein complexes, a predicted complex without a match in this reference dataset can still be a true protein complex. Therefore, for precise prediction of protein complexes, we have to consider if interacting

proteins (with complementary DDIs) can appear in the same cellular compartment. More precisely, if the predicted protein complex is located in multiple compartments, then either one of the members is a transmembrane protein or we consider it as false positive. As a further advancement, we tested the score after removing those complexes that are composed of proteins located in more than 2 compartments. To understand whether a complex composition is indeed consistent with the cellular compartment where its components were shown to be localized, we first checked what is the fraction of proteins of each protein complex are assigned in the same compartment in the CYC08 dataset [2]. About 80% of protein complexes show a fraction of 1, meaning that all their proteins are localized in the same compartment. Therefore, we removed predicted complexes which proteins are not in the same compartment. In the case without considering compartments the scores were reduced by 0.1 and 0.14, while in the case with compartments only by 0.01. Finally, we checked if other methods could achieve a higher score after removing complexes formed by proteins that are not located in the same compartment. ClusterOne [3] – one of the best tools available to predict protein complexes, but without quantitative information – had a score of 2.17 (0.21 lower than our best composite score), however once complexes not in the same compartment are removed, it drops down to 1.76 which is 0.37 lower than our best prediction.

1.5 Algorithm to merge similar complexes and reach Refined Complexes

The merging step requires to measure all pairwise similarity score for each pair of complexes, and this can be highly memory demanding when it needs to be performed for every SCs. SiComPre 1.0 simulations generate a large number of SCs more than 100,000 with human data), therefore to reduce the number of complexes and therefore the amount of memory required for this step, we first run this heuristic algorithm to merge similar complexes.

Next, we compute a similarity matrix where element *i,j* represents the overlap between complex *i* and *j*.

While the maximum overlap in the similarity matrix is greater than 0.5, we take the pair with the maximum overlap and we merge them, then we recompute the overlap for the resulting new

complex. Finally, we get the new maximum and we repeat the process. However, in this final step the overlap is not considered as we considered so far, but we increase the overlap linearly with the size of complexes merged.

$$\mathrm{OverlapNew}(c1, c2) = \mathrm{Overlap}(c1, c2) \times \left(\frac{(a - 4)}{(100 - 4)} \times 2.5 + 1\right)$$

Where *a* is the product of the complex sizes, length(*c1*) and length(*c2*), while the other parameters have been chosen arbitrarily to reduce as much as possible the number of redundant complexes. The meaning of this step is to let larger protein complexes to bind each other with a smaller overlap compared to small complexes. We want this because large complexes have a higher number of variants compared to smaller one, moreover they tend to have a large number of attachments with a smaller fraction of proteins in the core of the complex.

The maximum threshold of 0.5 has been considered because for small complexes we believe that protein complexes with smaller overlap are different enough to be considered two separated complexes, e.g. three protein long complexes that share two proteins have an overlap of 0.44 and we want to keep them separated, in the same time we want to have large complexes that share a smaller fraction of the proteins to be merged together.

The algorithm used to merge complexes is illustrated below:

```
mthr=max(0.9,Thr)
while mthr>=0.5 and mthr>Thr:
for id1,s1 in enumerate(complexes):
        for id2,s2 in enumerate(complexes):
                if id1<id2:
                        if len(s1)>0 and len(s2)>0:
                                if len(s2)/len(s1)>=mthr:
                                        if float(float(len(s1.intersection(s2))**2)
                                        /float(len(s1)*len(s2)) ) >=mthr:
                                                tmp=set(list(s1)+list(s2))
                                                if len(tmp)<150:
                                                        complexes[id1]=tmp
                                                        complexes[id2]=[]
                                else:
```

```
                        break

complexes_new=[]
for c in complexes:
        if len(c)>0:
                complexes_new.append(c)
complexes=complexes_new
mthr=max(mthr-0.1,Thr)
```

where *complexes* is a list of protein complexes ordered by size and *Thr* is the overlapping threshold used for merging.

1.6 Compare simulation results from different conditions

The algorithm used to compare results builds a network where each node is a protein complex in a given condition, edges represent the overlap between the two nodes (complexes). This network is clustered with MCL in order to find group of complexes that are similar among the different conditions (e.g. the same complex in two conditions will be clustered together). Finally, the sum of protein complex abundances within each cluster for each condition is used to determine the abundance of the given cluster in the given condition. These can be used to evaluate any difference between protein complexes in different condition. Quantitative variation will be shown as a difference between the abundance of the cluster in different condition; complexes with different proteins (qualitative variation) will not be clustered together resulting again in clusters that have different abundance (i.e. 0 in a condition and a positive number in the other condition).

2. Additional results

2.1 Performance with reduced protein abundances

SiComPre can be slower than existing methods to predict protein complexes since it requires to perform simulations with multiple copies of each proteins considering multiple binding sites and a large number of sub-volumes. This combinatorial problem can generate about 100,000,000 possible interactions. Some users might not have access to a machine equipped with the

necessary hardware to run such a complex simulation. Therefore, we provide the score of the qualitative prediction in yeast based on reduced protein abundances and without compartments (**Supplementary Table 4**). We keep at 3 the abundances of those proteins which would fall below 3 by this transformation. Again, protein complexes after refinement step (RC) were compared to CYC08 [2], this time without removing complexes that are not consistent with protein localization. Despite a good qualitative prediction, the refined complexes quantitative predictions cannot be reliable when protein abundances are reduced to low values.

3. Step-by-step tutorial to execute SiComPre 1.0

In this part, we are going to describe the 6 simple steps to predict protein complexes with SiComPre 1.0. Here we are going to use a simple model that can be reproduced with the data available in the DATA directory of SiComPre 1.0 package. It can be also loaded directly under EXAMPLES in the FILES menu. Other, more complex examples can be also directly imported the same way. These can be used to reproduce the results presented in the main text.

3.1 Step 1. Requirements

SiComPre can be executed on Windows and Linux without any hardware or software requirements. The presented example can be simulated on almost any state of the art computers, however proteome-wide models used in this study needs a lot of memory (up to 4-8 GB) and require a many computation hours. Therefore, it is strongly suggested to accelerate the computation using GPU computing and this requires a CUDA device (https://developer.nvidia.com/cuda-gpus) and updated graphics card drivers.

To run the whole pipeline from command line it is also necessary to have Python (only version 2.7 have been tested) with numpy package installed. This is provided as part of our installation package.

3.2 Step 2. Define compartments

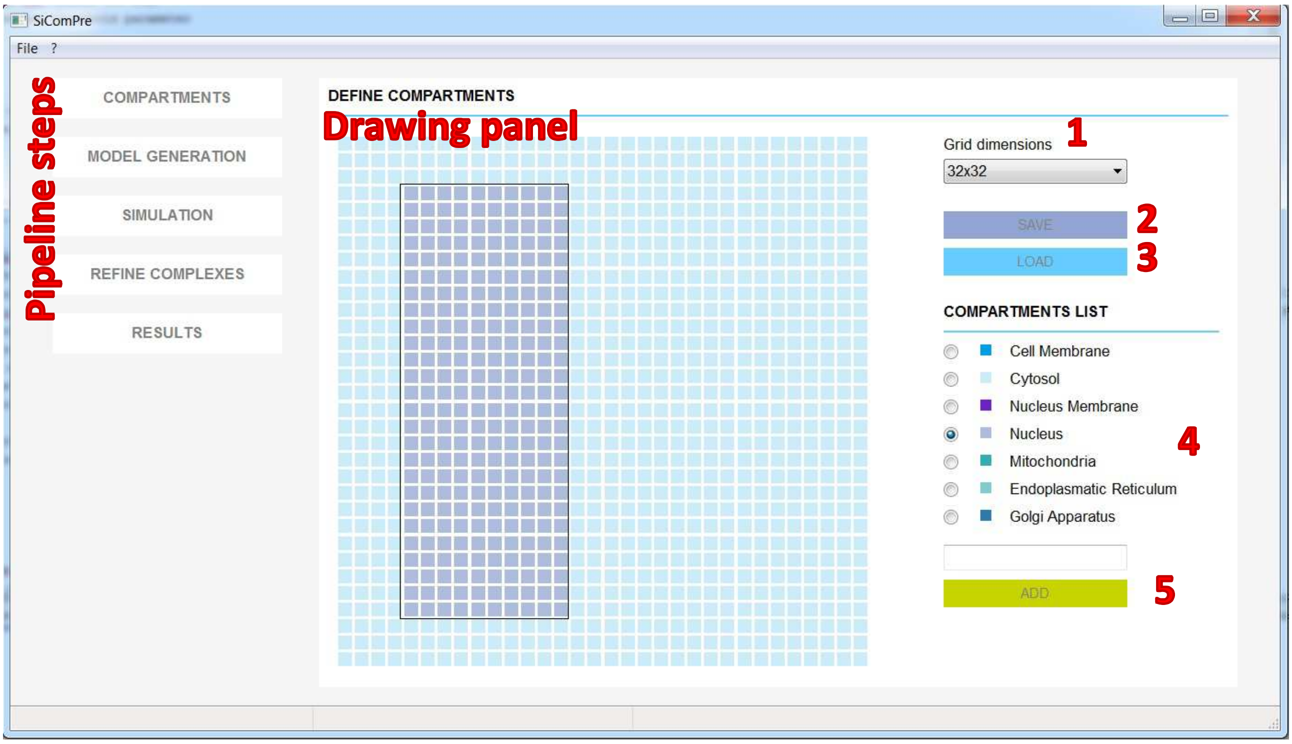

In this step it is possible to design or import the compartment organization for the simulation. Users can first select the grid size (1), save the current compartment model for later usage (2) or load previous model (3). The default model used in this study is provided with SiComPre package and can be loaded through (3). Saved (exported) models have the following format.

Compartment1:svID1;svID2; … ; svIDn;

CompartmentM:svIDx;svIDy; … ; svIDz;

Once imported, SiComPre will visualize the space.

Selecting the desired compartment one can assign lattices to a desired compartment (4) selecting cells in the drawing panel. It is also possible to define new compartments with button “Add” (5).

3.3 Step 3. Import input files and generate the model

The tool provides the option to load a few examples with all input files required. Here we explain how to modify these or add data from other input files.

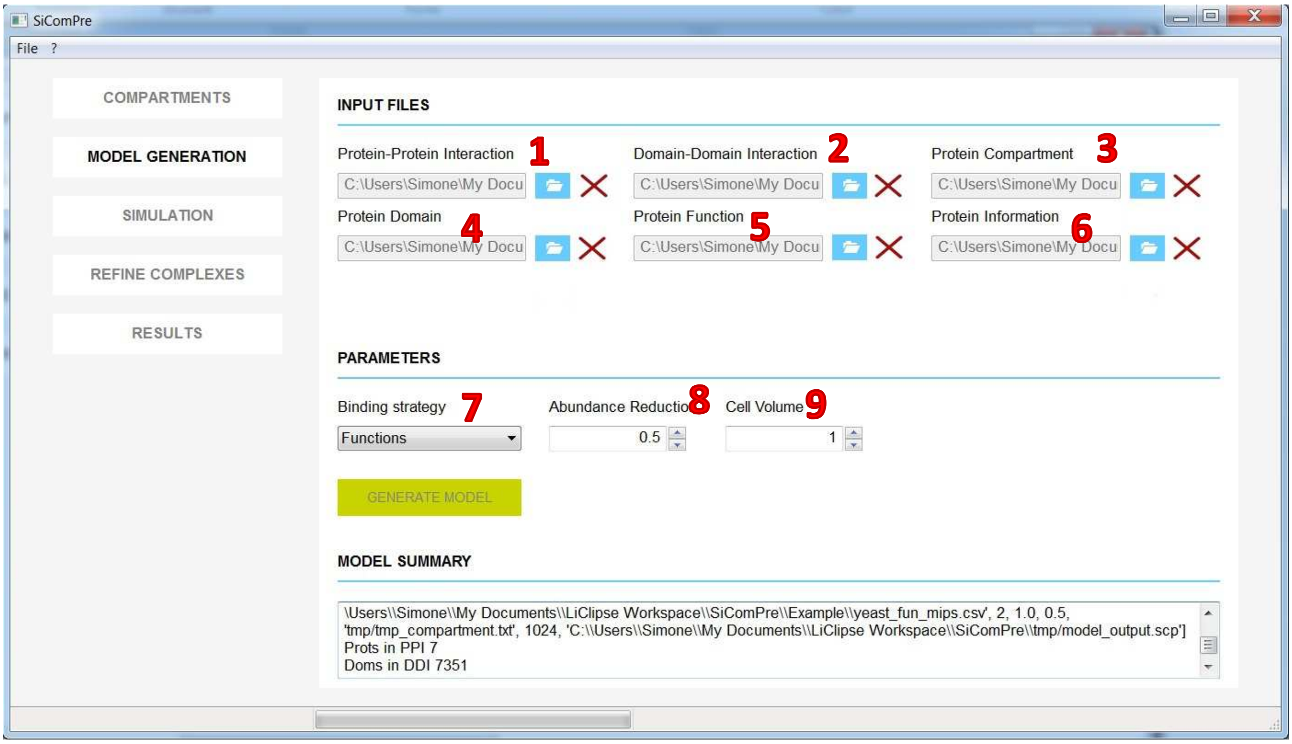

The tool requires to import the desired Protein-Protein Interactions dataset (1), Domain-Domain Interactions dataset (2), protein localizations (3), protein-domain associations (4), protein-function associations (5), protein information (abundances, diffusion rates, binding limits) (6). These text files require the following format.

1. PPI file: *Protein1* {tabular} *Protein2 [{tabular} binding rate (decimal) {tabular} unbinding rate (decimal)]*
2. DDI file: *Domain1* {tabular} *Domain2*
3. Protein localization file: *Protein* {tabular} *Compartment*
4. Protein-domain file: *Protein* {tabular} *Domain*
5. Protein-function file: *Protein* {tabular} *Function*
6. Protein information file: *Protein1* {tabular} *Abundance (decimal) [{tabular} diffusion rate (decimal) {tabular} binding limit (integer)]*

Fields *Protein*, *Domain* and *Compartment* are string and should not contain underscores '_'. Values in [] are optional and, if omitted, default values are used (diffusion rate = 1, binding limit=10). Check example files in the paragraph "Run a test".

Protein localization file (3) can be omitted if the no model for compartmentalization is used.

Three binding strategies are available as described in the related publication and in our previous study [4]:

- Full: If a DDI is not found between interacting proteins of a PPI then specific fictitious interacting domains are added to these proteins.
- noFictitious: A PPI is considered only if a corresponding DDI is found. This strategy yields less false positives, but the false negatives increase.
- Function: We add fictitious domains only if the proteins of the binary interaction are involved in the same biological function.

Protein abundances indicated in (6) are multiplied by cell volume (9) and elevated to the power of abundance reduction (8).

$$Model\ Protein\ Abundance = (FileProteinAbundance \times CellVolume)^{Abundance\ Reduction}$$

Therefore, if Abundance reduction is 0.5, the square root of the abundance will be used for the model.

Finally, users can be generated the model by clicking "GENERATE MODEL" button. A summary indicating the input files and the number of DDIs, PPIs and total number of proteins is displayed in the "MODEL SUMMARY" panel.

To further simplify the generation and execution of some models utilized in this paper, the tool allows to automatically import all the required files from the DATA directory of SiComPre. The models can be imported using the Load examples function in the File menu (see below).

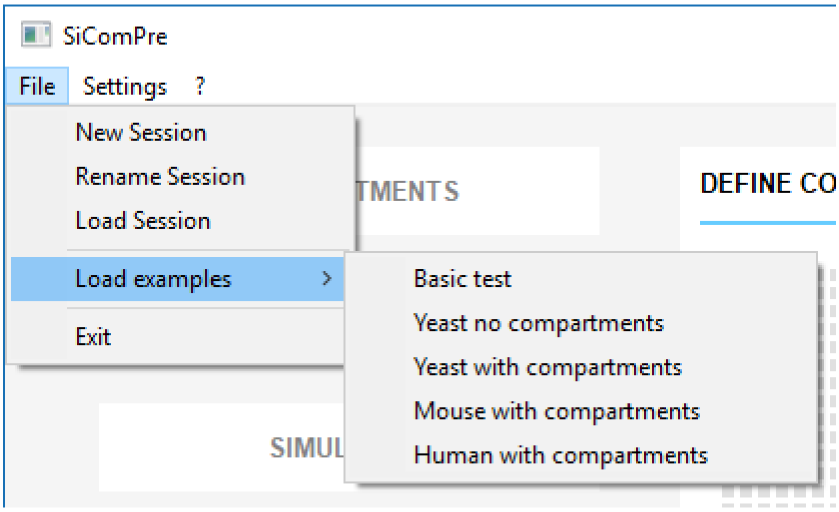

The basic test model load a demo example. This model can be executed with minimal computational requirements and it is useful to verify the correct installation of SiComPre.

The remaining models, requires more memory and hybrid GPU and CPU computation to be executed, however by reducing the abundance of the proteins in the simulation (parameter 8 and 9) it is possible to execute these models on a less powerful machine (see section 2.1).

3.4 Step 4. Run simulations

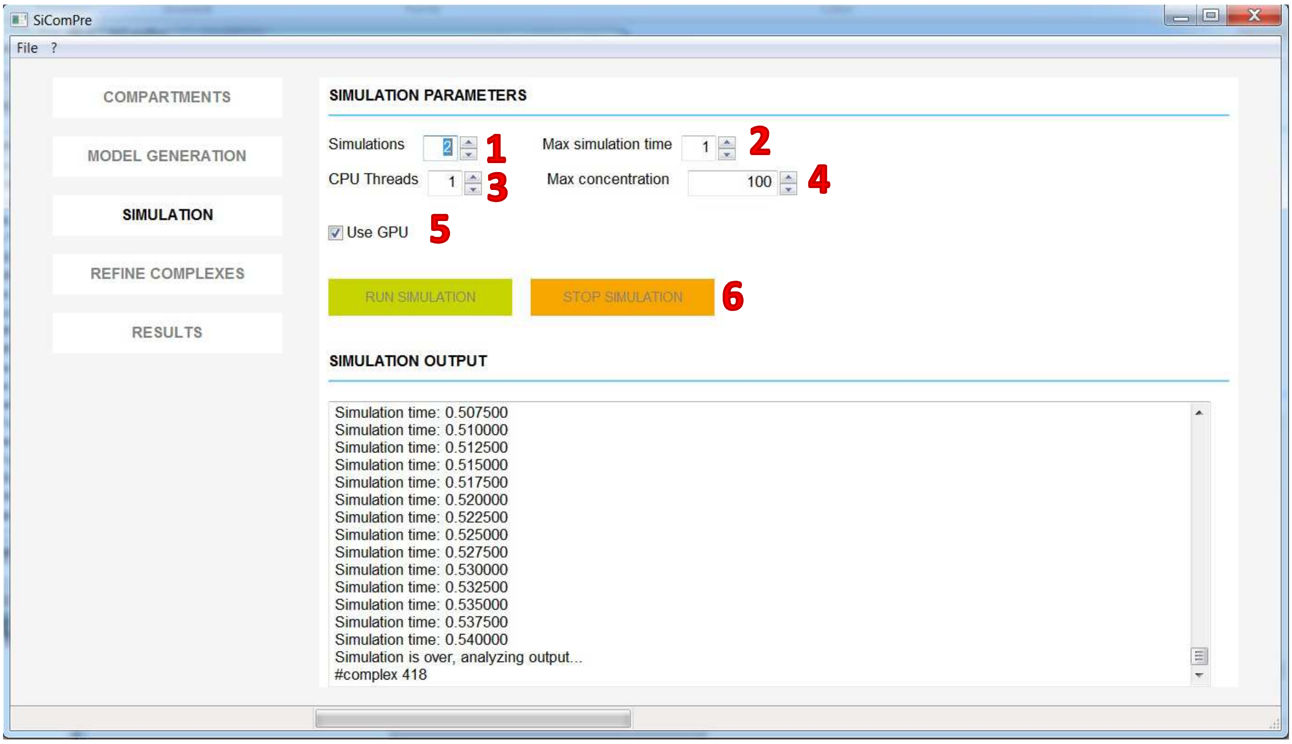

Now the model is ready to be simulated, select number of simulations to run (1), max simulation time (2) which is typically between 0.5 and 2.0, number of CPU threads to use (3) and the maximum number of molecules that can be located in the same sub-volume at the same time (4). Flag GPU option (5) to accelerate the computation with GPU; it requires NVIDIA graphics card and updated drivers. Click start to run the simulations. The button “STOP SIMULATION” (6) stops the simulation between the current and the next simulation. The process might take few seconds for model containing a small number of protein, but it requires up to one day for model of the human proteome. Time varies with the used hardware.

Simulations results are stored in a folder inside the session directory. The name of the folder is a positive integer indicating the number of simulation (one folder for each simulation). Output files are of three types:

- Trajectory_Molecule[*simulation_time*].out: contains the position of each protein in the grid at time ‘*simulation_time*’.
- resultCOMPLEX[*simulation_time*].out: stores the simulated protein complexes identified at time ‘*simulation_time*’. If ‘*simulation_time*’ is not indicated the file refers to the results at the end of the simulation.
- Complex structure[*simulation_time*].out contains all the binary interaction between proteins and domains at time ‘*simulation_time*’. If ‘*simulation_time*’ is not indicated the file refers to the results at the end of the simulation.

3.5 Step 5. Refine complexes

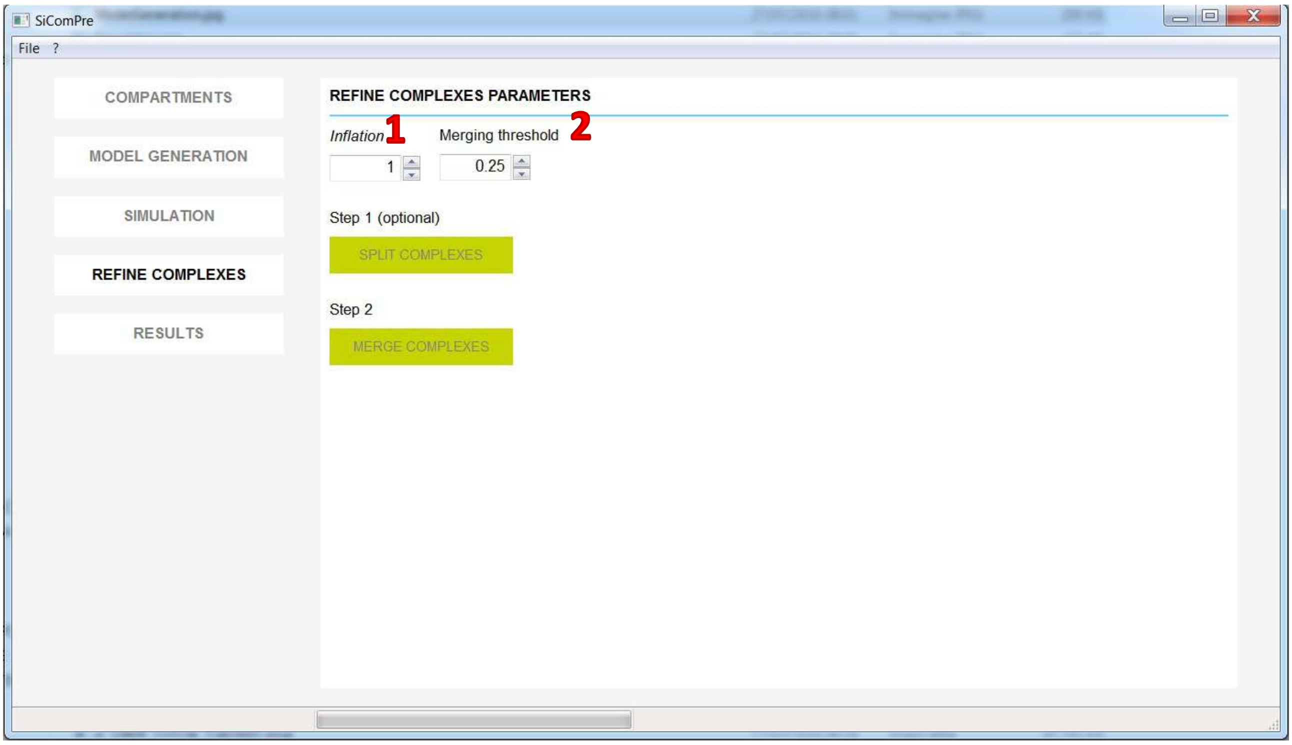

In this part users can execute the steps needed to refine protein complexes as explained in the main article. Users only need to define the inflation parameter used by Markov Clustering (1) and the merging threshold (2) used to merge similar complexes. Markov Clustering with high inflation will break a complex in more pieces compared to a smaller value. The higher is the merging threshold the larger will be the generated protein complex. This process will be applied to each simulation in the session directory and resulting protein complexes are stored in session_directory/simulation_directory/refined_complexes. Finally, all refined complexes in different simulations will be merged together with the same algorithm in a single file located at session_directory/refined_complexes.txt

3.6 Step 6. Check and export results

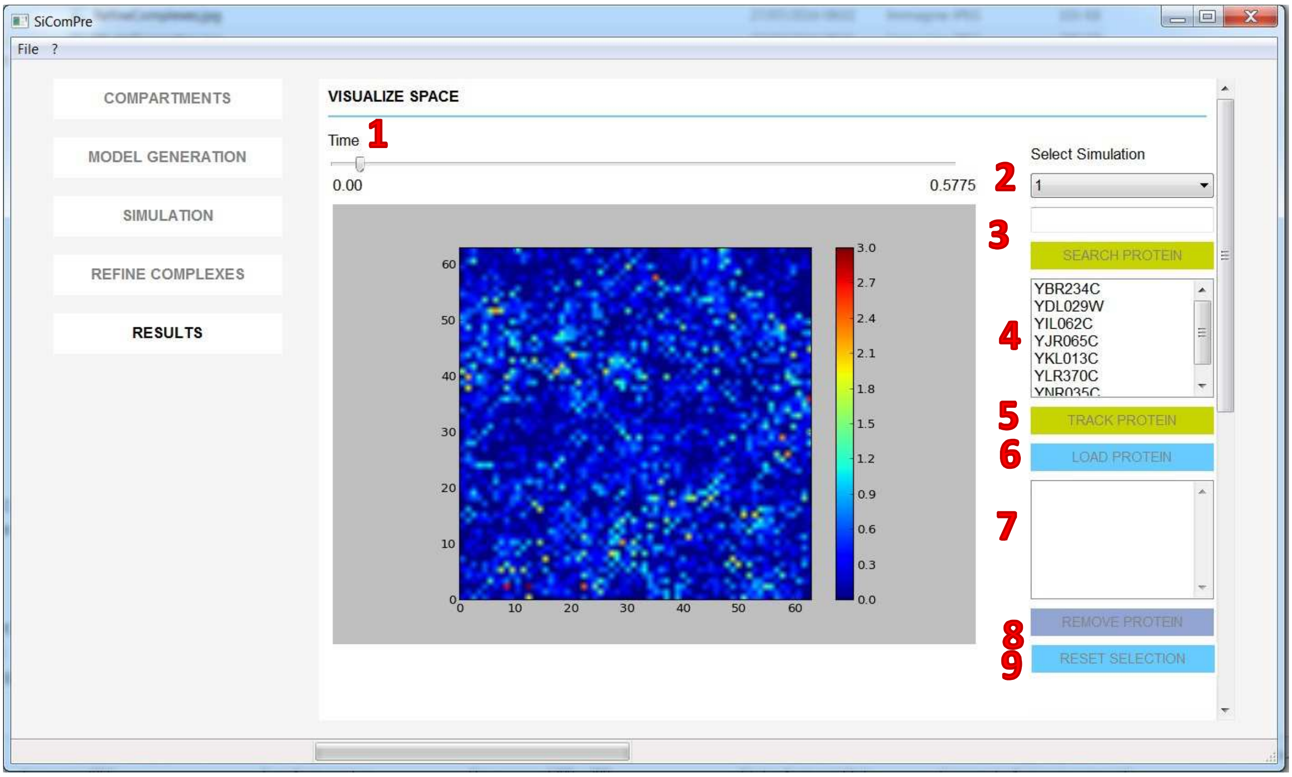

Users can visualize the distribution of protein in the simulation space and how they diffuse over time (1). This is done for each simulation that can be selected with (2). It's also possible to restrict the visualization to a smaller set of proteins, listed in the textbox (7). It's possible to add proteins selecting them from the list of available proteins (4) and click "TRACK PROTEIN" (5) or, if users want to add multiple proteins without selecting them one by one, it is possible to load a file containing a list of proteins to be tracked (6). In this file, each protein name needs to be written in a different line. Through search panel (3) users can find search for protein in the list (4). Buttons (8-9) allow to remove proteins from (7).

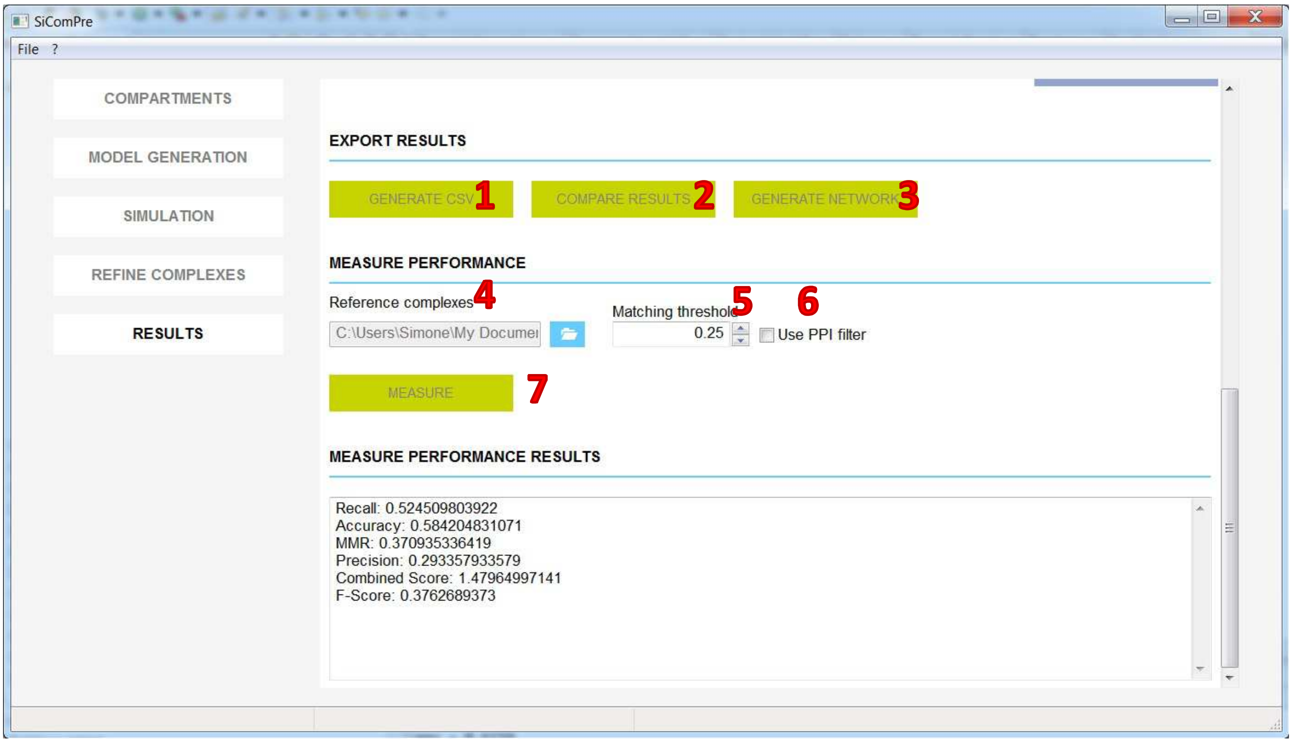

Results are now ready to be exported and analyzed with other tools. CSV files, generated clicking button (1), can be imported in software such as MS Excel where it is possible to visualize the predicted protein complex abundance among the simulations, known complexes matching a predicted complex and the link to DAVID analysis chart for the involved proteins. Once terminated, the file will be available in the session directory with the name “prediction.csv”. When comparing results (2), users need to select the directory of a previous session containing the same number of simulations, next the algorithm described in the main text will be executed. Results are stored in the session directory. Users can also generate a network of a specific complex by defining the ID indicated in the “prediction.csv” file generate with button (1). Network files can be imported in Cytoscape to generate networks like those used in this manuscript (3). However, it is not possible to generate the network of complexes that shares at least one common protein as their relative network would be merged together in Cytoscape. Finally, it is possible to compare the qualitative prediction to a set of reference complexes loaded with (4). Matching threshold (5) is used as minimum overlapping score necessary to match a reference complex. PPI filter (6) filters out reference complexes that were not represented in the PPI file used to generate the model.

3.7. Save and Load sessions

SiComPre assign to each session a directory that contains all results and file generate through the pipeline. When a new session is started, the default directory is SICOMPRE_PATH/tmp. Each simulation creates a sub-directory named with the simulation number. Users can control session through the File menu.

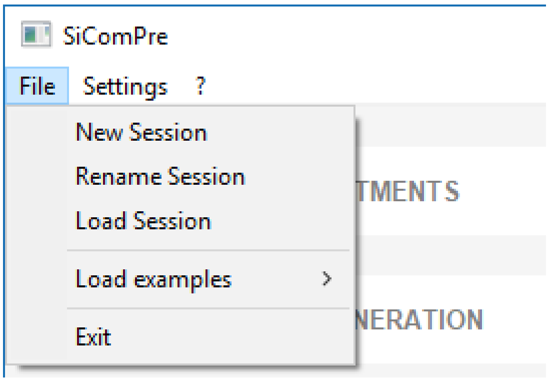

Renaming the session will rename the directory “tmp” with a different name. This will prevent to be overwritten by a new session. To load a previous session, users need to select the directory of a previous session, in this way SiComPre will change the session directory to the one selected by the user.

3.8. Execute SiComPre from the command line

For more expert users, it is possible to execute SiComPre directly from the command line with the python script runPipeline.py

```
python runPipeLine.py PPI DDI ProteinDomain ProteinAbundance Compartments ProteinFunction Strategy(1-3) CellVolume AbundanceReduction #SV ModelName MaximumConcentration ReferenceComplexes MergingThr TimePoints #Simulations
```

Users can modify this Python script to have a personalized pipeline execution.

In case of multiple GPUs in the system, users can indicate the ID of the GPU device to be used by editing the line

GPU_ID = 0

In the runPipeLine.py. ID=0 usually indicate the default GPU. It is possible to disable GPU computation by setting this value to -1.

3.9. Run a test

To run a simple test at the scope to test the installation, we provided some example files located in the “Data/Example” directory (**Supplementary Table 6**). Execution time of this test should take only few seconds. “Protein – Location association” and “Compartment model” can be omitted. A more realistic test can be performed using the file “collins2007rRNA.txt” and tweaking the `CellVolume` value in order to run a simulation with a small or high number of proteins. Alternatively, it is possible to use the “load example -> basic test” function in the file menu to automatically import a demo model. More complicated models used in this paper can be imported too, but they will require more computational power to be executed.

3.10. Utils provided as separate scripts

We also provide some scripts useful to convert external database file such as Biogrid [5], MOPED [6], Pax-DB [7] or Gene Ontology (GO) to a tabular format that can be read by SiComPre.

Conversion from GO compartments

The convertCompartments.py script converts protein localization associated to GO terms to organelles (e.g. mitochondria and integral to membrane are converted to “cm” which stands for cytoplasm mitochondria membrane). All localization related to complexes, such as ribosome, are deleted.

Execution: `python convertCompartments.py GO_compartments`

The script uses as input a tab separated file download from GO database containing two columns. The first one indicates the protein ID and the second one the name of the compartment.

Conversion from MOPED

The abundanceMOPED.py script converts files exported from MOPED database to the right format needed for SiComPre.

Execution: `python abundanceMOPED.py file_downloaded_from_moped condition`

The first parameter is the filed downloaded from moped, and the second parameter is the condition used to filter proteins (column 9 of the input file).

Conversion from Pax-DB

The abundancePAXDB.py script converts files exported from PAX-DB database to the right format needed for SiComPre. Typically, protein ID on PAX-DB is different from Uniprot which is used in the example data provided with SiComPre, thus it is necessary to provide a mapping file to convert protein IDs.

Execution: `python abundancePAXDB.py mapping_file file_downloaded_from_paxdb`

Where mapping file contains two columns separated with a tab. The first column indicates the ID of the protein in the Pax-DB database and the second column the protein IDs to be used in the output file.

4. Selection of the best PPI for human studies

SicomPre 1.0 works with context-specific information; therefore it is natural to ask if we should use a context-specific PPI (e.g. a tissue-specific PPI). In a context-specific network we have binary interactions that are observed only in particular cell type or condition. However, if some interaction is observed only in specific tissues it is probably due an emergent behavior arises from the different abundance levels. Instead, with SiComPre 1.0 we want to use PPI as a context-free source, containing interactions where proteins that are in the proximity of each other can interact. Using protein abundances, instead, we will limit the possibility of protein interactions. Therefore,

the behavior of context-specific interactions will be intrinsically embedded in the simulations. Under this idea, we need to have a PPI that reflects multiple conditions, increasing the probability to identify protein interactions that are possible to observe only in specific tissue (e.g. protein X is expressed only in liver).

In this study, we aim to have the highest possible coverage of the human proteome, therefore we want to test multiple PPI available in the literature in order to find the one that can better predict protein complexes from a reference dataset [8].

We tested different PPIN with different coverage, for these experiments we run our simulation using Pancreas and B cells protein abundances which are the cell type and tissue among those with the highest number of protein expressed in the Kim et al. experiments [9], therefore yielding to more reliable results. To make our findings not dependent purely on SiComPre we also tested these PPI with ClusterOne [3].

As shown in **Supplementary Table 5**, Hippie with a threshold of 0.8 shows a better coverage with the second best precision. LitBM13 also reaches similar results. Indeed, in none of these networks we observed important complexes such as Ribosomes and Proteasomes. A more precise network has been proposed by Havugimana et al. [10] containing 13992 interactions, however the coverage is very limited, probably because it has been generated from specific cell lines where many interactions are improbable to appear. In this dataset, we observed nearly all the important complexes, but many other were missing leading to a poor recall. However, what is important for us is also the ability of predicting important complexes such as ribosome and proteasome.

We didn't find any correlation between the number of PPIs or proteins covered with the Recall; suggesting that high ratio of FP interactions not only leads to a low precision but could influence the formation of complexes which proteins are covered by the PPI.

As a results of this analysis, we used Hippie [11] with 0.8 as threshold for the prediction of protein complexes in human.

5. Additional steps for reproducibility

5.1. Protein complexes localization

Protein localization are extracted from GO database with the following query

```
SELECT Distinct D.xref_key, T.name FROM ( (go_latest.association AS A JOIN go_latest.term AS T ON A.term_id=T.id) JOIN go_latest.gene_product AS P ON A.gene_product_id=P.id ) JOIN dbxref AS D ON P.dbxref_id=D.id WHERE T.term_type='cellular_component' AND P.species_id='363592' AND D.xref_dbname='UniprotKB';
```

For mouse the ID used is 275242 which is associated with Mus Musculus, while molecular functions are retrieved changing the field 'cellular_component' with 'molecular_function'.

GO cellular components contain also protein complexes that might bias the results. These are deleted with:

```
DELETE FROM human_compart WHERE compart LIKE '%complex%';
```

ID mappings are performed with UniProt IDmapping webtool. In case of multiple mapped IDs the interaction/protein has been duplicated.

5.2 Estimate protein-rRNA interactions and rRNA abundances

We were not able to find protein-rRNA interactions for eukaryotic ribosome. To solve this issue, we download the tertiary structure of the eukaryotic ribosome at 3.0 A of resolution from the Protein Data Bank (PDB ID: 4V88). Using Rasmol – a tool for molecular visualization – we identify all the proteins that are in contact with the different rRNAs (25S, 5.8S, 5S and 18S). The Rasmol script is the following:

```
select within(3.0, :A) #A is the chain corresponding to one of the rRNA molecule
```

```
show selected chain
```

where A is the chain corresponding to a rRNA molecule.

In this way, we selected all the chains (proteins or other rRNA) which have at least one group within 3.0 A of distance from the desired rRNA molecule. We chose 3.0 A, because the length of all molecular bounds in ribosome should be shorter than 3.0 A. Therefore, this process gives us a good approximation of which proteins are in contact with the rRNA. Next, since ribosome is well conserved among eukaryotic organisms, we found all the homologous protein in Homo Sapiens.

The second step is to estimate the number of rRNA in a cell. Again, since we know that ribosome and rRNA is well conserved among eukaryotic organisms, we used the amount of rRNA in a Yeast cell which is 1870 PPM.

Finally, we manually added a function (called rRNAint) to each protein in the PDB file and to the rRNA. This will generate a pair of interacting fictitious domain for each protein interacting with rRNA.

For mitochondrial ribosome, instead, we used interactions data derived from prokaryotic ribosome, according the endosymbiosis theory, and we identify the corresponding homologous in Homo Sapiens. MrRNA abundance has been estimated as (#rRNA in proka * number of mitochondria in Yeast / number of proteins in yeasts) * 1,000,000. The number of mitochondria per cell has been obtained from Bionumbers (http://bionumbers.hms.harvard.edu//bionumber.aspx?id=108845&ver=2).

6. References:

1. Alberts BJ, Lewis A, Raff J. Molecular biology of the cell2008.

2. Pu S, Wong J, Turner B, Cho E, Wodak SJ. Up-to-date catalogues of yeast protein complexes. Nucleic Acids Res. 2009;37(3):825-31. doi: 10.1093/nar/gkn1005. PubMed PMID: 19095691; PubMed Central PMCID: PMC2647312.

3. Nepusz T, Yu H, Paccanaro A. Detecting overlapping protein complexes in protein-protein interaction networks. Nature Methods. 2012;9(5):471-2.

4. Rizzetto S, Priami C, Csikasz-Nagy A. Qualitative and Quantitative Protein Complex Prediction Through Proteome-Wide Simulations. PLoS Comput Biol. 2015;11(10):e1004424. doi: 10.1371/journal.pcbi.1004424. PubMed PMID: 26492574.

5. Chatr-Aryamontri A, Breitkreutz BJ, Heinicke S, Boucher L, Winter A, Stark C, et al. The BioGRID interaction database: 2013 update. Nucleic Acids Res. 2013;41(Database issue):D816-23. doi: 10.1093/nar/gks1158. PubMed PMID: 23203989; PubMed Central PMCID: PMC3531226.

6. Kolker E, Higdon R, Haynes W, Welch D, Broomall W, Lancet D, et al. MOPED: Model Organism Protein Expression Database. Nucleic Acids Res. 2012;40(Database issue):D1093-9. doi: 10.1093/nar/gkr1177. PubMed PMID: 22139914; PubMed Central PMCID: PMC3245040.

7. Wang M, Weiss M, Simonovic M, Haertinger G, Schrimpf SP, Hengartner MO, et al. PaxDb, a Database of Protein Abundance Averages Across All Three Domains of Life. Molecular & Cellular Proteomics. 2012;11(8):492-500. doi: DOI 10.1074/mcp.O111.014704. PubMed PMID: WOS:000308024200021.

8. Ruepp A, Waegele B, Lechner M, Brauner B, Dunger-Kaltenbach I, Fobo G, et al. CORUM: the comprehensive resource of mammalian protein complexes-2009. Nucleic Acids Research. 2010;38:D497-D501. doi: Doi 10.1093/Nar/Gkp914. PubMed PMID: WOS:000276399100080.

9. Kim MS, Pinto SM, Getnet D, Nirujogi RS, Manda SS, Chaerkady R, et al. A draft map of the human proteome. Nature. 2014;509(7502):575-81. doi: 10.1038/nature13302. PubMed PMID: 24870542.

10. Havugimana Pierre C, Hart GT, Nepusz T, Yang H, Turinsky Andrei L, Li Z, et al. A Census of Human Soluble Protein Complexes. Cell. 2012;150(5):1068-81. doi: http://dx.doi.org/10.1016/j.cell.2012.08.011.

11. Schaefer MH, Fontaine JF, Vinayagam A, Porras P, Wanker EE, Andrade-Navarro MA. HIPPIE: Integrating protein interaction networks with experiment based quality scores. PLoS One. 2012;7(2):e31826. doi: 10.1371/journal.pone.0031826. PubMed PMID: 22348130; PubMed Central PMCID: PMCPMC3279424.

12. von der Haar T. A quantitative estimation of the global translational activity in logarithmically growing yeast cells. BMC Syst Biol. 2008;2:87. doi: 10.1186/1752-0509-2-87. PubMed PMID: 18925958; PubMed Central PMCID: PMC2590609.

13. Jackson DA, Pombo A, Iborra F. The balance sheet for transcription: an analysis of nuclear RNA metabolism in mammalian cells. FASEB J. 2000;14(2):242-54. doi: 10.1096/fj.1530-6860. PubMed PMID: 10657981.

14. Lee TI, Young RA. Transcription of eukaryotic protein-coding genes. Annu Rev Genet. 2000;34:77-137. doi: 10.1146/annurev.genet.34.1.77. PubMed PMID: 11092823.

15. Adam SA. The nuclear pore complex. Genome Biol. 2001;2(9):REVIEWS0007. doi: 10.1186/gb-2001-2-9-reviews0007. PubMed PMID: 11574060; PubMed Central PMCID: PMC138961.

16. Deng C, Xiong X, Krutchinsky AN. Unifying fluorescence microscopy and mass spectrometry for studying protein complexes in cells. Mol Cell Proteomics. 2009;8(6):1413-23. doi: 10.1074/mcp.M800397-MCP200. PubMed PMID: 19269952; PubMed Central PMCID: PMC2690482.

17. Mavrich TN, Ioshikhes IP, Venters BJ, Jiang C, Tomsho LP, Qi J, et al. A barrier nucleosome model for statistical positioning of nucleosomes throughout the yeast genome. Genome Res. 2008;18(7):1073-83. doi: 10.1101/gr.078261.108. PubMed PMID: 18550805; PubMed Central PMCID: PMC2493396.

18. Collins SR, Kemmeren P, Zhao X-C, Greenblatt JF, Spencer F, Holstege FC, et al. Toward a comprehensive atlas of the physical interactome of Saccharomyces cerevisiae. Molecular & Cellular Proteomics. 2007;6(3):439-50.

19. Kim Y, Min B, Yi GS. IDDI: integrated domain-domain interaction and protein interaction analysis system. Proteome Sci. 2012;10 Suppl 1:S9. doi: 10.1186/1477-5956-10-S1-S9. PubMed PMID: 22759586; PubMed Central PMCID: PMC3380739.

20. Ghaemmaghami S, Huh W-K, Bower K, Howson RW, Belle A, Dephoure N, et al. Global analysis of protein expression in yeast. Nature. 2003;425(6959):737-41.

21. Dongen Sv. Graph Clustering by Flow Simulation [PhD thesis, University of Utrecht]2000.

22. Tatsuke D, Maruyama O. Sampling strategy for protein complex prediction using cluster size frequency. Gene. 2013;518(1):152-8. Epub 2012/12/14. doi: 10.1016/j.gene.2012.11.050. PubMed PMID: 23235119.

23. Liu G, Wong L, Chua HN. Complex discovery from weighted PPI networks. Bioinformatics. 2009;25(15):1891-7. doi: 10.1093/bioinformatics/btp311. PubMed PMID: 19435747.

24. Bader GD, Hogue CW. An automated method for finding molecular complexes in large protein interaction networks. BMC Bioinformatics. 2003;4:2. PubMed PMID: 12525261; PubMed Central PMCID: PMC149346.

25. Zaki N, Efimov D, Berengueres J. Protein complex detection using interaction reliability assessment and weighted clustering coefficient. BMC Bioinformatics. 2013;14. doi: Unsp 163

Doi 10.1186/1471-2105-14-163. PubMed PMID: WOS:000320127300001.

7. Supplementary Figures and Tables

Simulation Results	With Compartments	Without Compartments	
	SV=4096	SV=1024	SV=4096
BL=10	2.17	2.23	2.32
BL=100	2.14	2.24	2.32

Supplementary Table 1. Parameter tuning for different binding limits and sub-volumes on simulated complexes.

Pipeline Steps	With Compartments		Without Compartments	
	Split	No Split	Split	No Split
Simulation (BL=10, SV=4096)	2.17	2.17	2.32	2.32
Split	2.25	-	2.38	-
Merging	2.14	2.09	2.24	2.18
Consistent with cellular compartments	2.13	2.08	2.10	2.08

Supplementary Table 2. Assessment of qualitative prediction through the SiComPre 1.0 pipeline. SiComPre pipeline consists of different steps (simulation, split, and merging); for each of these steps, we reported composite score after each step including and not including compartments. Furthermore, we tested SiComPre 1.0 without splitting complexes. Finally, we filtered out complexes that are not consistent with cellular compartments and reported the composite score after each of these steps.

Protein complex	Experimental abundance	SiComPre prediction with Compartments	SiComPre prediction with Compartments and RNA	Average of constitutive subunit abundances	Experimental Evidences
Ribosome	187,000	96,928	6615	53,331	An average of 187,000 copies has been estimated [12].
RNA polymerase I	1,500	3,383	11	5,031	Assuming the proportion of RNA polymerases in Mus Musculus[13] is maintained in yeast, the abundance is the half of that of RNA polymerase II.
RNA polymerase II	3,000	19	58	4,375	An average of 3,000 complexes has been estimated in Yeast [14].
RNA polymerase III	150	0	152	3,352	Similary for RNA polymerase I, approximated to be 1/20 of that of RNA polymerase II
Nucleopore Complex	200	295	373	2,120	An average of 200 NPC has been estimated [15].
Eisosome	75	53,824	205	109,500	It is the average of Eisosomes in yeast [16].
Nucleasome	57,000	98,073	128	267,640	The estimated number of nucleosomes in yeast [17].
Anaphase - Promoting Complex	3,000	1469	18	494,2	There are about 1000-5000 APC per cell in yeast [16].
Mitochondrial ribosomes	5,685	1951	1296	2815,36	http://bionumbers.hms.harvard.edu/bionumber.aspx?&id=107351&ver=2&trm=fraction%20ribosomes%20mitochondrion
Pearsons' Correlation		0.77	0,94	0.28	

Supplementary Table 3. Comparison of SiComPre quantitative prediction. This table has been extended from our previous study. Only a few protein complex abundances are available in

the literature. We summarized these, providing also a short explanation of how these were estimated. We compared SiComPre predictions against the trivial method of predicting protein complex abundances using the average of the abundance of the constitutive subunits. SiComPre predictions show a better agreement to experimental data compared to predictions based on the protein abundance averages.

	Abd ^ 0.5	Abd ^ 0.0625	Abd ^ 0 + 2
Composite Score	2.10	2.10	2.03
Recall	0.82	0.82	0.78
MMR	0.59	0.59	0.57
Accuracy	0.70	0.70	0.68

Supplementary Table 4. Effects of the reduction in protein abundances. Yeast proteome models were generated with reduced protein abundances and SIComPre 1.0 composite scores were calculated for three different reduction methods. In the first column, we checked SiComPre 1.0 with the square root of protein abundances. In the second one we further reduced the protein copies in the simulation by elevating each protein abundances to the power of 0.0625. Lastly, we considered only 3 copies for each protein in the model.

Dataset	Proteins covered	Enriched Compartments	Enriched Tissue	PPIs	Density	SiComPre			ClusterOne		
						**Recall**	**Precision**	**MMR**	**Recall**	**Precision**	**MMR**
**Havigiumana** [10]	3006	Lumens<br>Ribonucleoproteins<br>Non-membrane<br>Cytosol	Epithelium<br>Fetal brain cortex<br>Cajal-Retzius cell<br>Platelet<br>Cervix carcinoma	13992	**0.001549**	0.1324	0.0783	0.0950	0.05	**0.05**	0.01
**Lit BM 13**	5545	Cytosol<br>Organelles and nuclear lumen<br>Non-membrane<br>Membrane parts	Epithelium<br>Platelet<br>Cervix carcinoma<br>Placenta<br>T-cell<br>Fetal brain cortex	11046	0.000359	0.3733	0.1209	0.2233	0.03	0.03	0.01
**Hi II 14**	4303	Cytosol<br>Organelles and nuclear lumen<br>Non-membrane	Skin<br>Uterus<br>Lung	13945	0.000753	0.0826	0.0318	0.0497	0.00	0.01	0.00
**Hippie (0.7)** [11]	**9570**	Membrane<br>Non-membrane<br>Nucleus<br>Cytosol	Epithelium<br>Platelet<br>Placenta<br>Cervix carcinoma	**49016**	0.000535	0.3495	0.0866	0.2099	**0.11**	0.04	**0.03**
**Hippie (0.8)** [11]	5639	Membrane<br>Non-membrane<br>Nucleus<br>Cytosol	Epithelium<br>Platelet<br>Placenta<br>T-cell<br>Cervix carcinoma	16806	0.000529	**0.3925**	0.1567	**0.2444**	**0.11**	0.04	**0.03**
**Hippie (0.9)** [11]	2436	Membrane<br>Non-membrane<br>Nucleus<br>Cytosol	Epithelium<br>Platelet<br>Placenta<br>T-cell<br>Cervix carcinoma	4131	0.000696	0.3654	**0.2175**	0.2152	**0.11**	0.04	**0.03**

Supplementary Table 5. Results in this table have been calculated based on CORUM dataset, without filtering any complex. LitBM13, Havugimana's PPI and Hippie tissue enrichment have a much lower p-value than those in hiII14. Hippie [11] (0.9 and 0.8) fails in predicting ribosome, but it can weakly predict proteasome. Hippie (0.7) [11] weakly predicts ribosome and proteasome (overlap score < 0.25). HiII14 fails in predicting both ribosome and proteasome. Similarly, clusterOne [3] cannot predict proteasome and ribosome with Lit BM 13 and HiII14, and the ribosome has a weak matching with Hippie. The number of simulation runs for this table is 4 for B cells, since the 5th is not improving the results and 2 for pancreas (3rd is not improving).

File Name	Input File Type	Description
ppi_arp23.csv	PPI file	Subset of the Collins PPI dataset [18] containing only proteins of the arp23 complex.
ddi_iddi.csv	DDI file	IDDI DDI dataset [19]
yeast_compartments.csv	Protein localization file	Yeast protein location from GO
yeast_protein_domains.csv	Protein–domain file	Yeast protein domain from GO
yeast_functions_mips.csv	Protein–function file	Yeast protein function from GO
yeast_abundances_ghe.csv	Protein information file	Yeast Protein abundances from Ghaemmaghami et al. [20]
compartments.csv	Compartment model	Example of compartment model used to generate results in this study. Load this in the "Compartments" screen.
reference_dataset_cyc08.csv	Reference Complexes	CYC08 reference complexes [2] needed to evaluate the results.

Supplementary Table 6. List of files to run SiComPre. This table provide the description of each file necessary to run SiComPre demo. These files can be automatically imported by clicking File -> Load examples -> basic test.

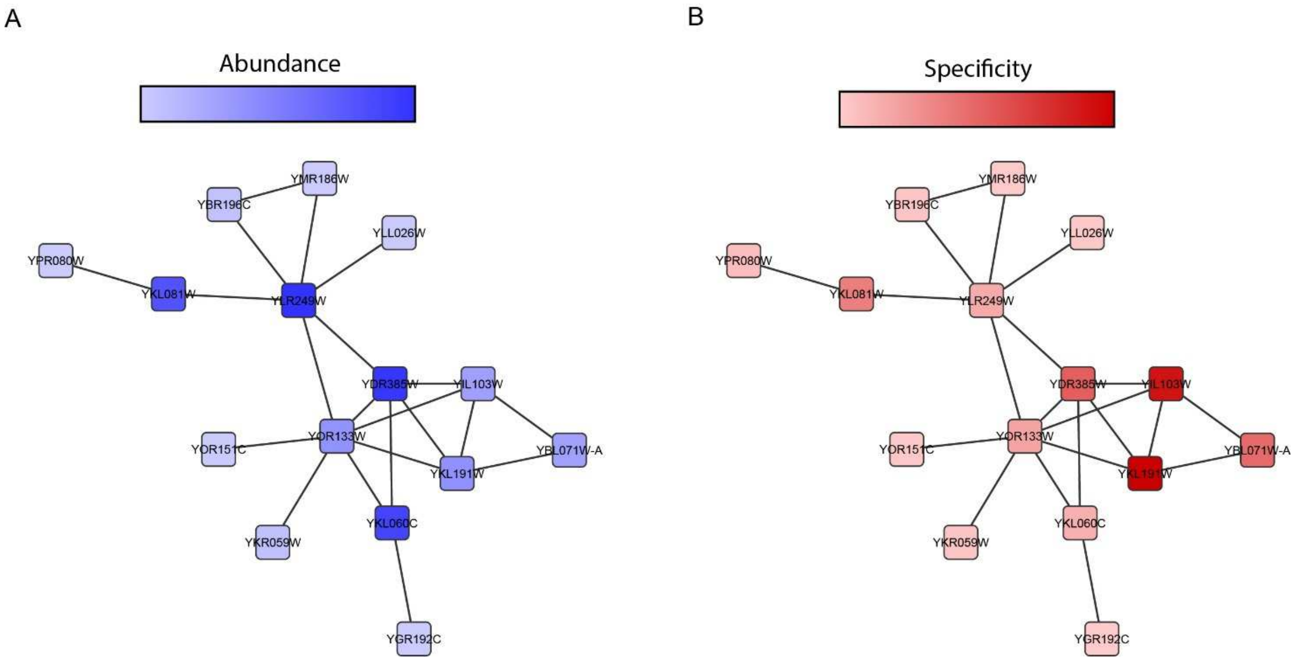

Supplementary Figure 1. Export network feature. These networks have been generated with the "export network" feature of SiComPre and represent the structure of a protein complex that does not have any match with reference complex dataset. Its components are enriched in translational regulation (elongation factors) and metabolic processes (proteins involved in glycolysis pathway, i.e. FBA-1, PGI1 and TDH3). Darkness represent the abundance of each protein in the complex (**A**) and their specificity in the complex (**B**).

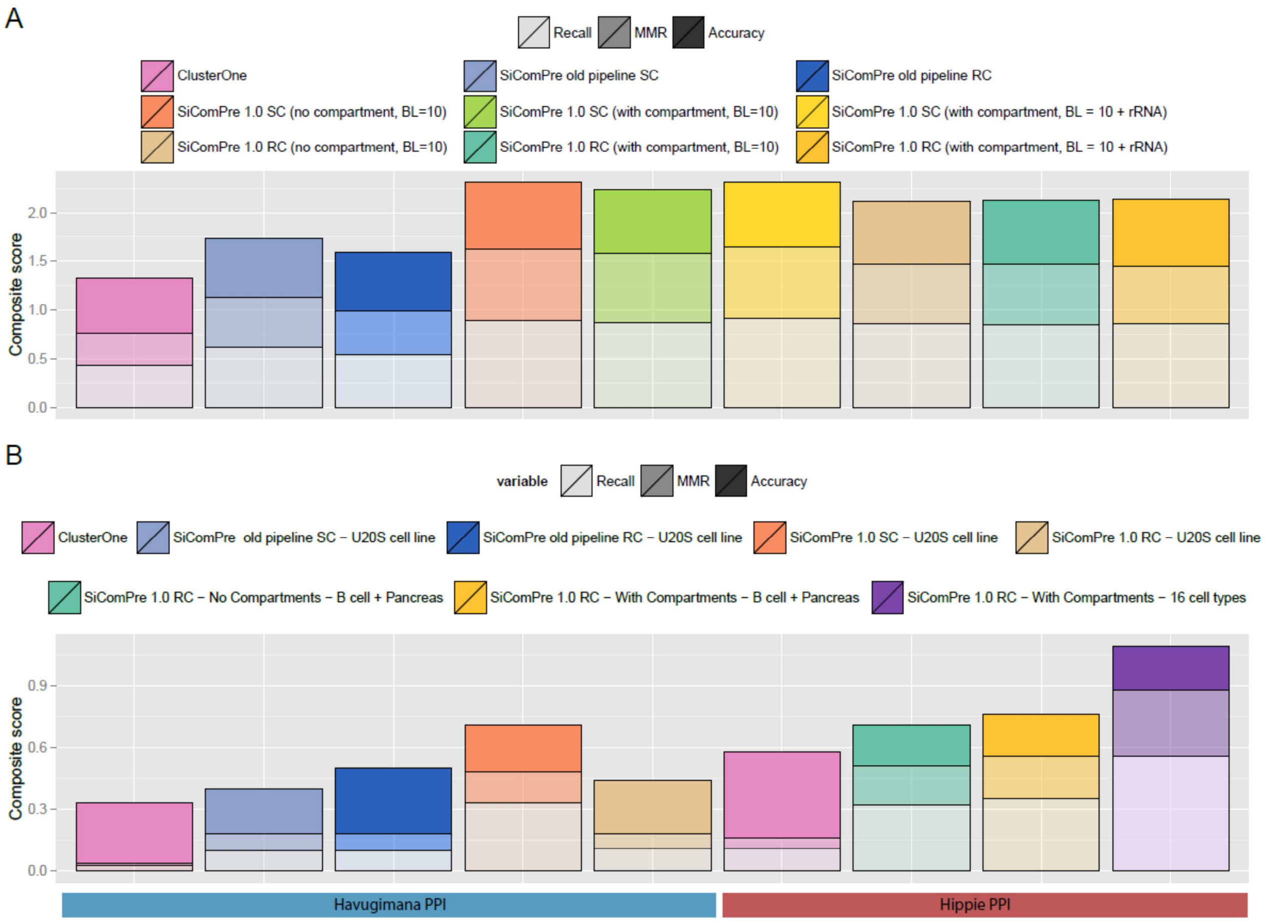

Supplementary Figure 2. Composite score obtained with different methods and different SiComPre 1.0 settings in Yeast and Human. Composite score was tested in Yeast against CYC08 reference dataset, Collins PPI dataset was used as input of SiComPre 1.0. ClusterOne and SiComPre 1.0 using the same settings used in the previous pipeline were tested as comparison using the same PPI dataset (**A**). To assess the quality of the prediction in Human, predicted complexes from ClusterOne and SiComPre 1.0 were compared to the CORUM reference dataset (**B**). Both Havugimana's and Hippie PPI datasets were tested. When tested with Havugimana PPI dataset [10], SiComPre 1.0 after simulation (SiComPre 1.0 SC) had a better composite score than the refined complexes (SiComPre 1.0 RC), this might be due to the number of complexes obtained after the simulation, which is between 10^5 and 10^6, about 100 times higher than RC.

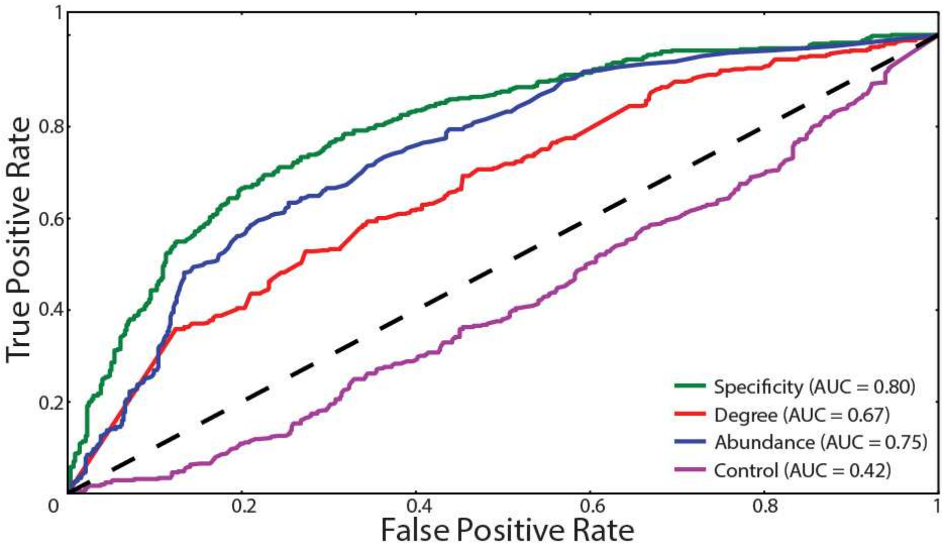

Supplementary Figure 3. AUC bait prediction. ROC curve to measure the score of various protein features extracted by our simulation results in predicting good baits for TAP-MS experiments.

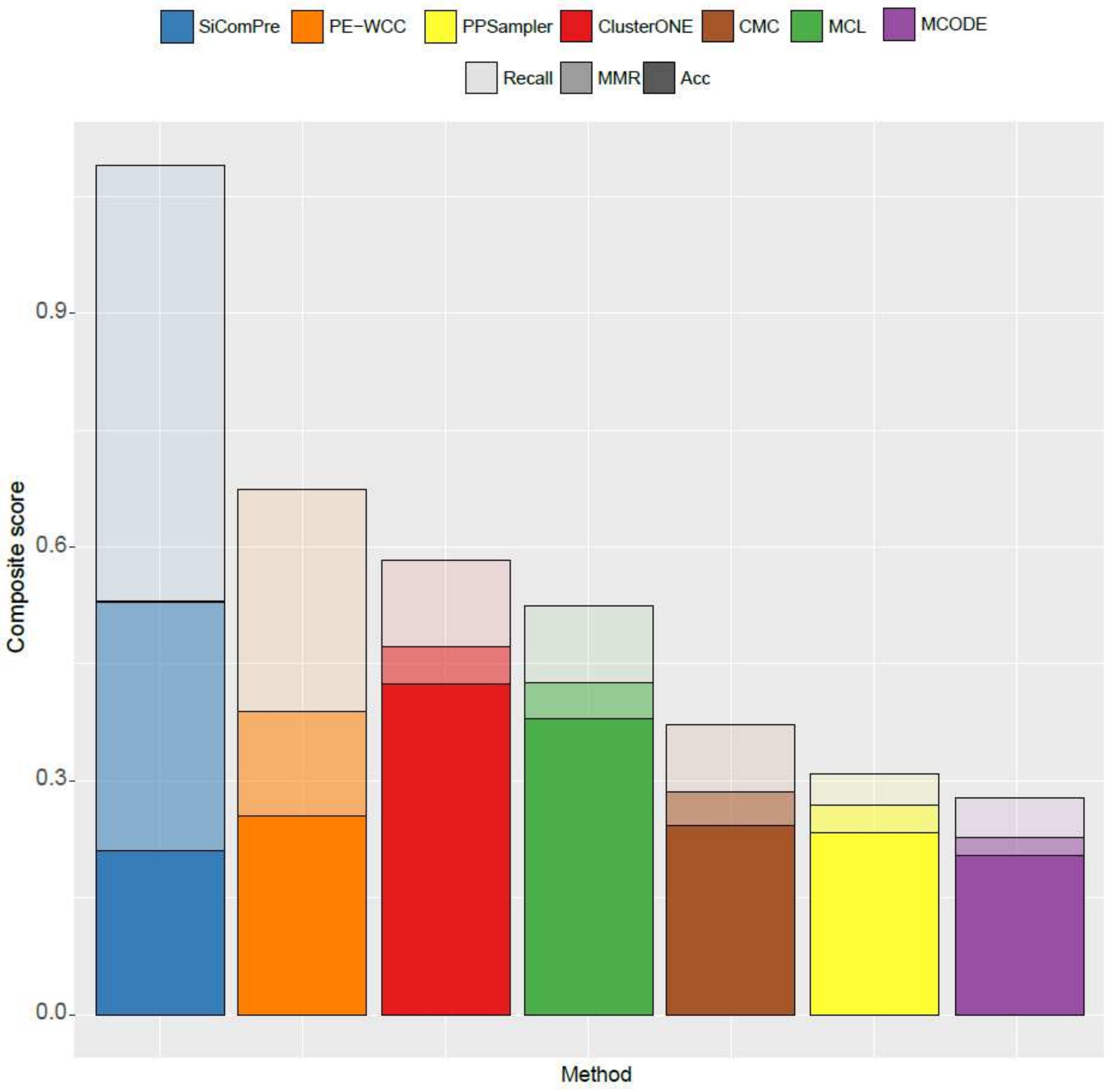

Supplementary Figure 4. Composite score obtained with different methods compared to human protein complexes. To assess the quality of the prediction in Human, predicted complexes from multiple methods [3, 21-25] were compared to the CORUM reference dataset using Hippie PPI. When methods allowed the use of PPI weights we used the full network, otherwise a filter was used to exclude low confidence interactions (weight > 0.8). SiComPre was executed using protein abundance from 16 cell types.

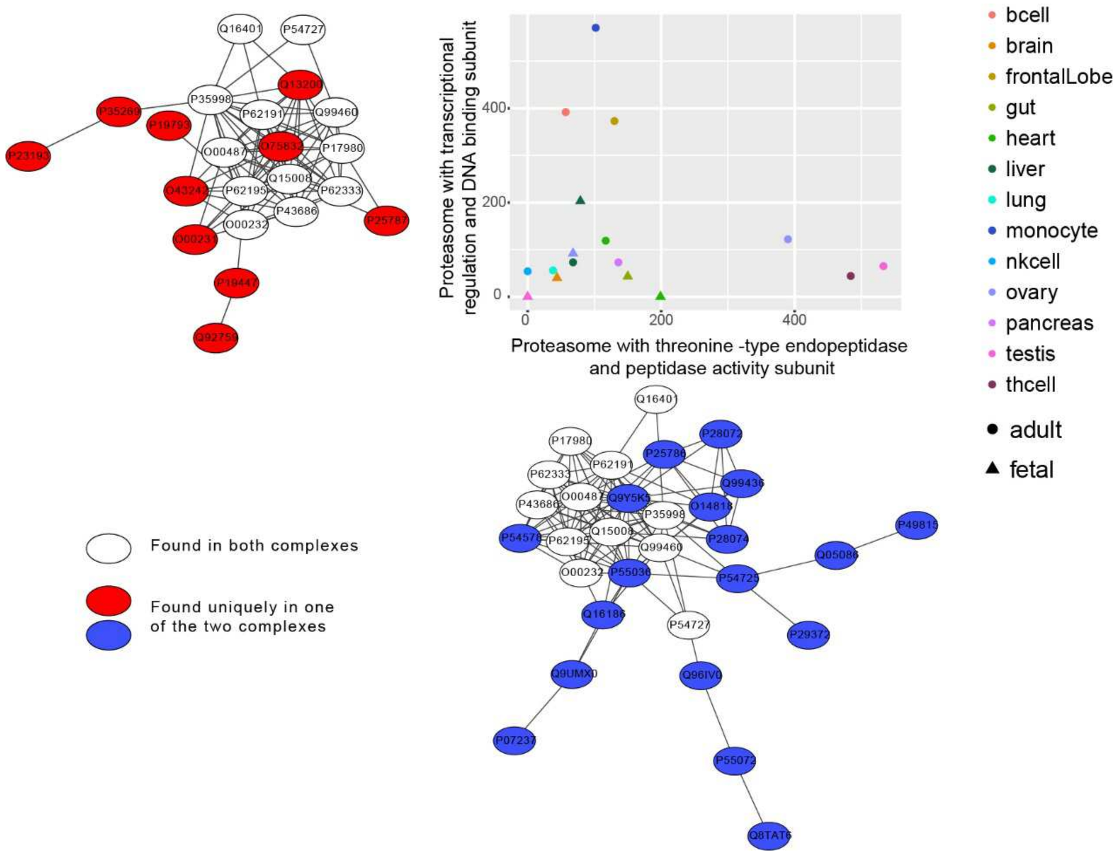

Supplementary Figure 5. Proteasome variation across human tissues and cell types. Quantitative variation of two proteasome subunits with its corresponding structure. Red nodes were protein found only in the subunit associated with transcriptomic regulation and DNA binding, while blue nodes are proteins identified only in the threonine-type endopeptidase and peptidase activity subunit. Protein occurrences in the first complex range from 1 to 214, while in the second one protein occurrences range from 1 to 1277.

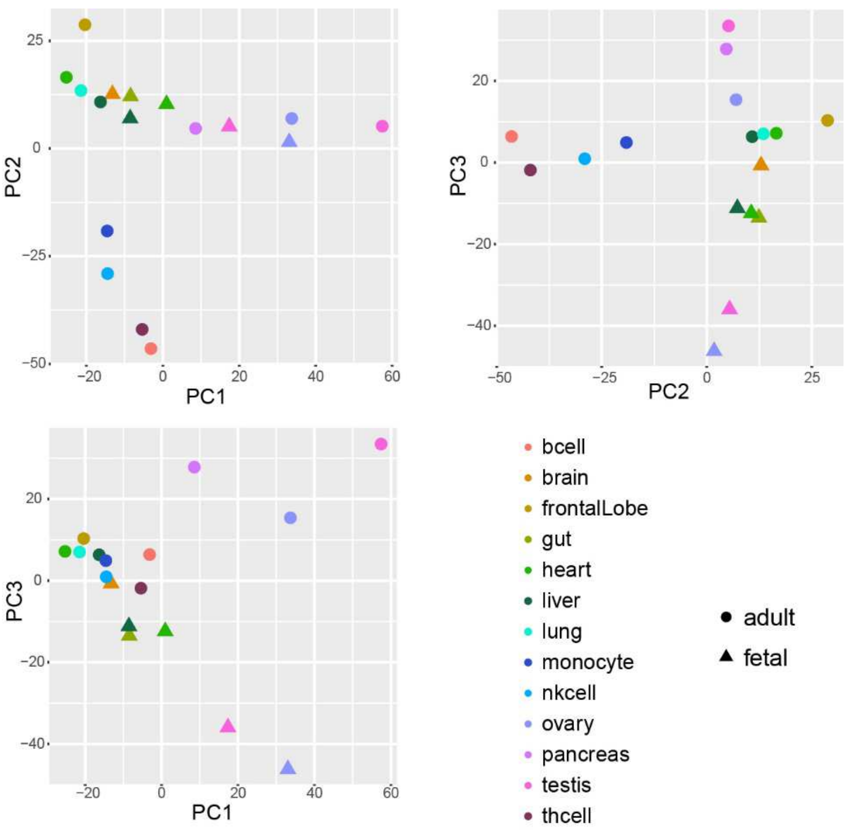

Supplementary Figure 6. Predicted protein complexes based PCA. Unsupervised principal component analysis separates reproductive tissues, immune system and fetal tissues cells.

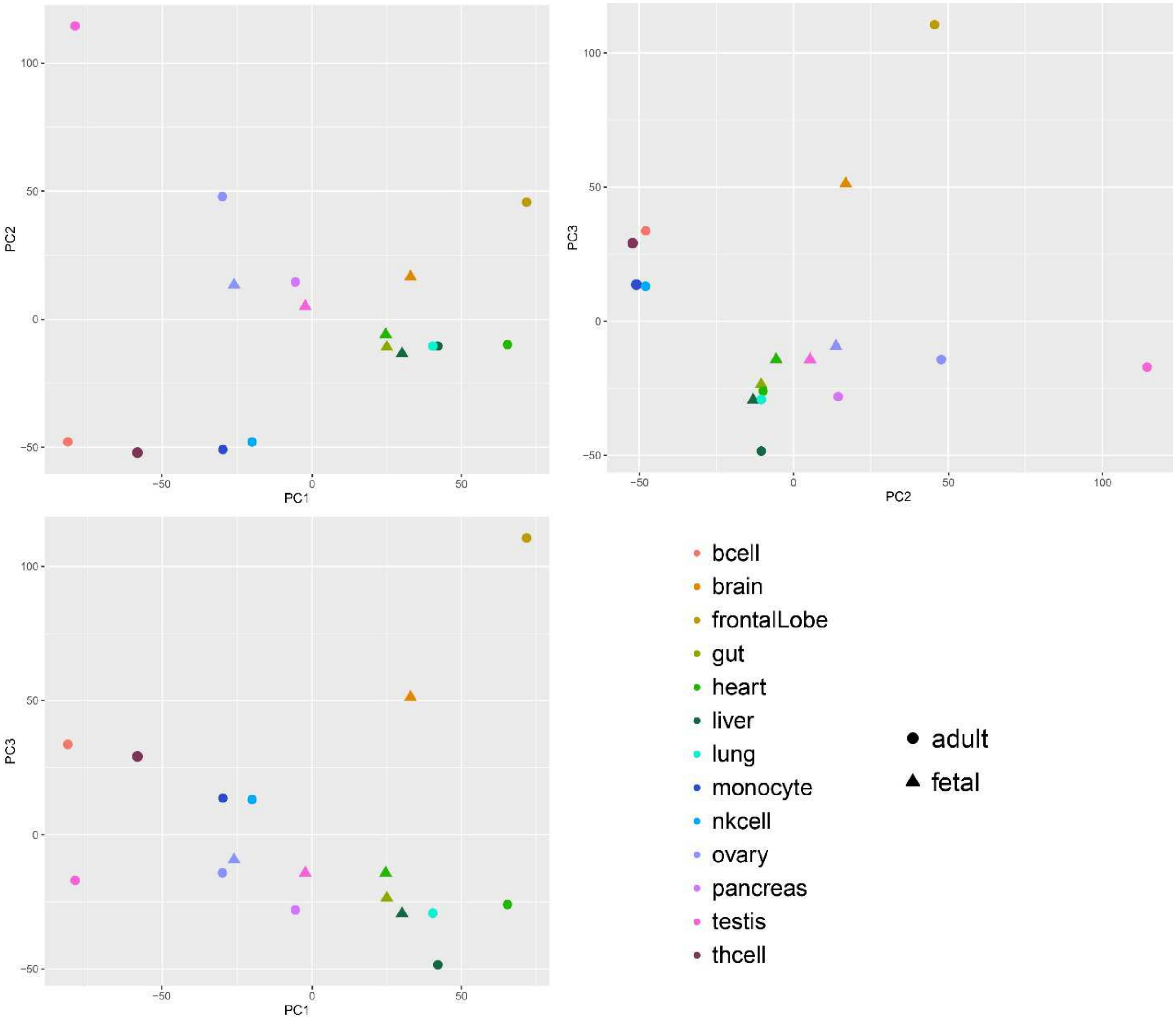

Supplementary Figure 7. Protein based PCA. Unsupervised principal component based on the protein abundances. Fetal and Adult tissues cannot be separated.

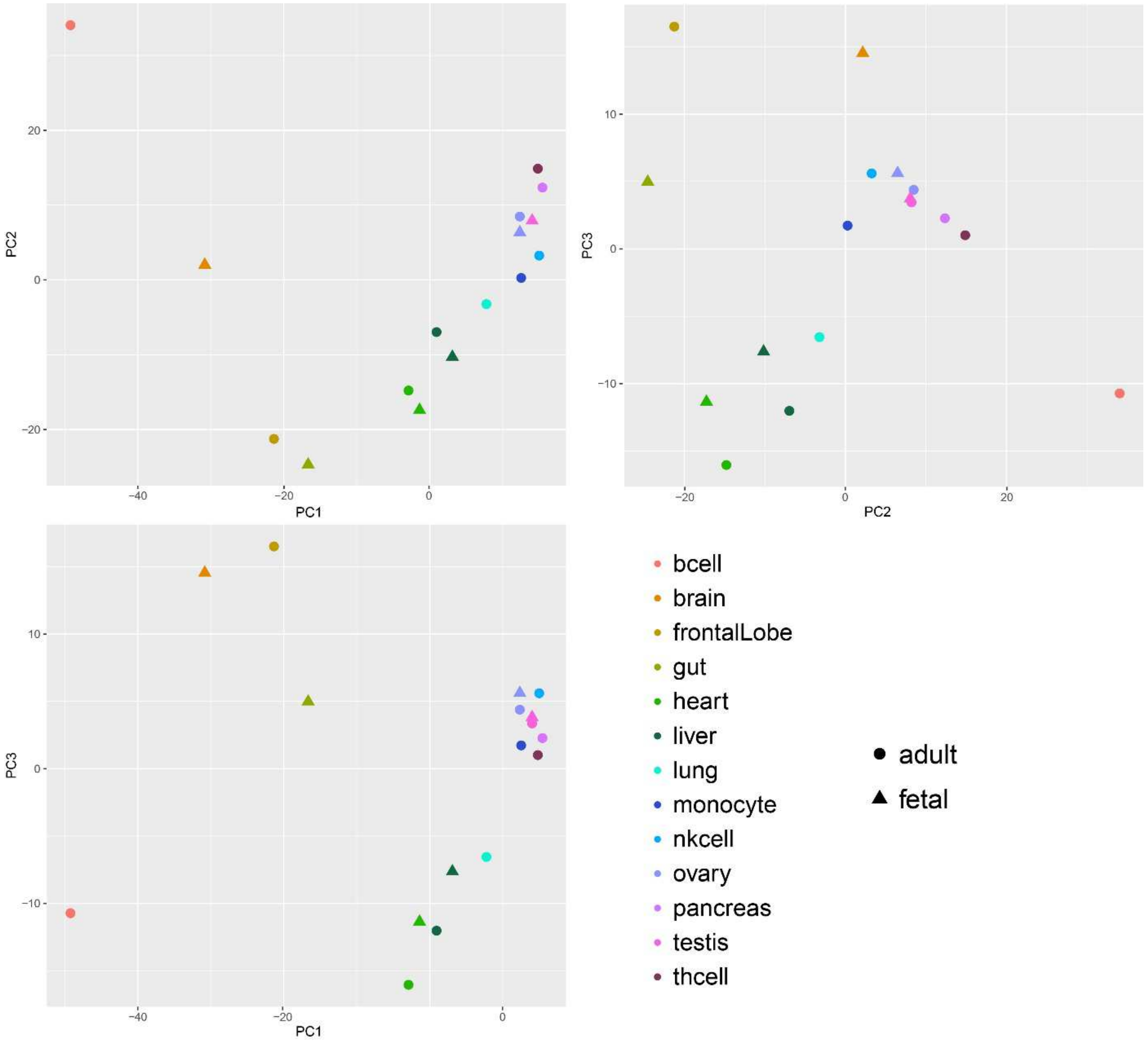

Supplementary Figure 8. CORUM protein complexes based PCA. Unsupervised principal component based on the average of protein abundance in a CORUM protein complex. Again, Fetal and Adult tissues cannot be separated.

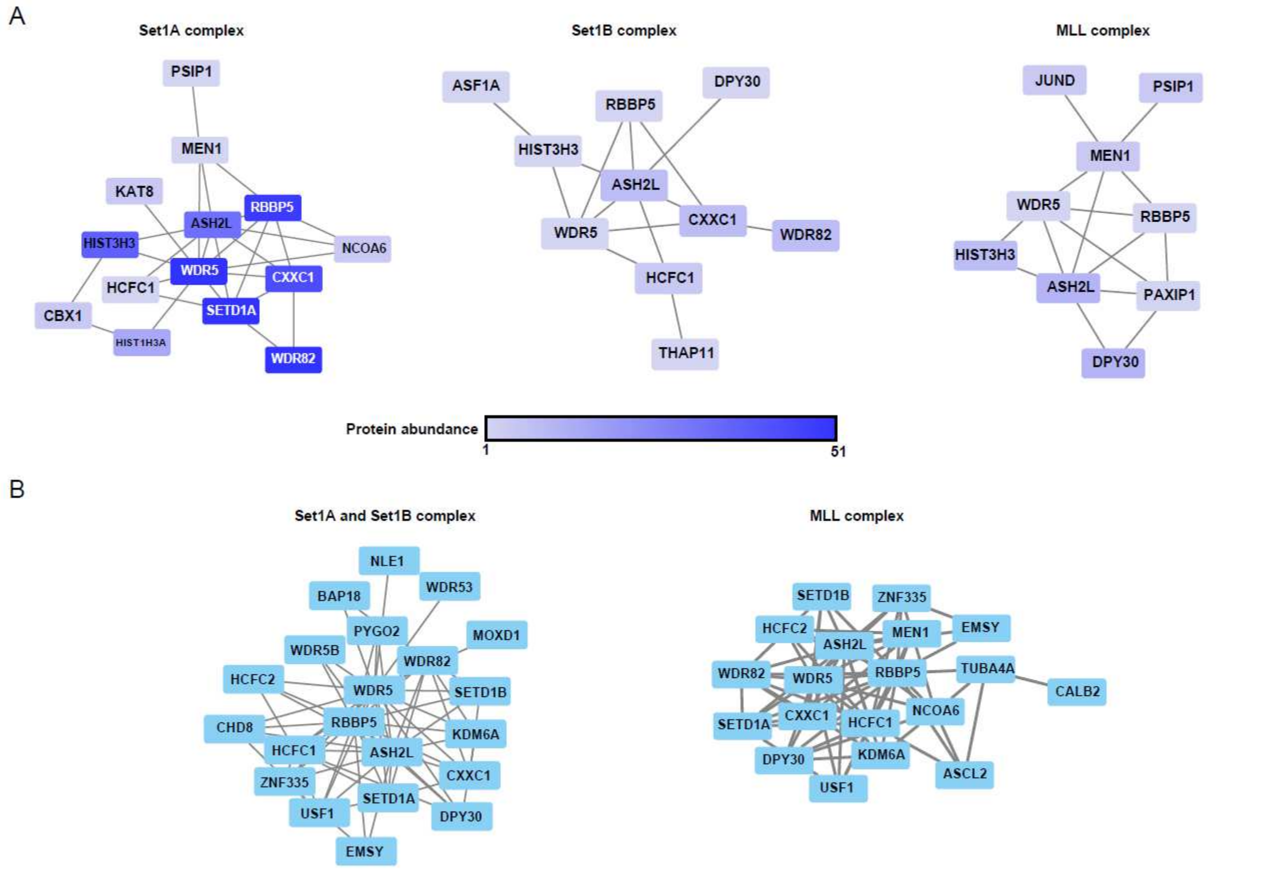

Supplementary Figure 9. SET/MLL protein complexes variants. The networks represent protein complex variants matching Set1A, Set1B, and MLL protein complex predicted by SiComPre (A) and clusterOne (B). For SiComPre prediction, color indicates how many times a protein appeared in a simulated complex matching the corresponding refined complex. As expected, Set1DA is highly abundant in the Set1A protein complex, but it is not identified in the other variants. ClusterOne could not differentiate between Set1A and Set1B protein complex, predicting the protein Set1DA and Set1DB as part of the same variant in both Set and MLL complex prediction.
